# Loss of glucocorticoid receptor phosphorylation contributes to cognitive and neurocentric damages of the amyloid-β pathway

**DOI:** 10.1186/s40478-022-01396-7

**Published:** 2022-06-22

**Authors:** Yann Dromard, Margarita Arango-Lievano, Amelie Borie, Maheva Dedin, Pierre Fontanaud, Joan Torrent, Michael J. Garabedian, Stephen D. Ginsberg, Freddy Jeanneteau

**Affiliations:** 1 Institut de Génomiqueénomique Fonctionnelle, Université de Montpellier, INSERM, CNRS, 34090 Montpellier, France; 2 Imagerie du Petit Animal de Montpellier, 34090 Montpellier, France; 3 Institut de Neuroscience de Montpellier, INSERM, 34090 Montpellier, France; 4 Department of Microbiology, New York University Grossman School of Medicine, New York, NY 10016 USA; 5 Nathan Kline Institute, Orangeburg, NY 10962 USA; 6 Departments of Psychiatry, Neuroscience & Physiology, NYU Neuroscience Institute, New York University Grossman School of Medicine, New York, NY 10016 USA

**Keywords:** Glucocorticoid receptor, BDNF, Memory, Neuroimaging, Spine dynamics

## Abstract

Aberrant cortisol and activation of the glucocorticoid receptor (GR) play an essential role in age-related progression of Alzheimer's disease (AD). However, the GR pathways required for influencing the pathobiology of AD dementia remain unknown. To address this, we studied an early phase of AD-like progression in the well-established APP/PS1 mouse model combined with targeted mutations in the BDNF-dependent GR phosphorylation sites (serines 134/267) using molecular, behavioral and neuroimaging approaches. We found that disrupting GR phosphorylation (S134A/S267A) in mice exacerbated the deleterious effects of the APP/PS1 genotype on mortality, neuroplasticity and cognition, without affecting either amyloid-β deposition or vascular pathology. The dynamics, maturation and retention of task-induced new dendritic spines of cortical excitatory neurons required GR phosphorylation at the BDNF-dependent sites that amyloid-β compromised. Parallel studies in postmortem human prefrontal cortex revealed AD subjects had downregulated BDNF signaling and concomitant upregulated cortisol pathway activation, which correlated with cognitive decline. These results provide key evidence that the loss of neurotrophin-mediated GR phosphorylation pathway promotes the detrimental effects of the brain cortisol response that contributes to the onset and/or progression of AD dementia. These findings have important translational implications as they provide a novel approach to treating AD dementia by identifying drugs that increase GR phosphorylation selectively at the neurotrophic sites to improve memory and cognition.

**Supplementary Information:**

The online version contains supplementary material available at 10.1186/s40478-022-01396-7.

## Introduction

Alzheimer’s disease (AD) is a worldwide public health issue impacting cognition and quality-of-life for millions of elders that cannot be currently cured, prevented, or treated efficiently. Aberrant processing, accumulation, and deposition of well-studied proteins amyloid-β precursor protein (APP) and Tau contribute to neurodegeneration, neural network malfunction and cognitive decline [14]. Amnesia is one of the early and progressive symptoms characterized by deficits of memory retrieval [47]. The memory trace is still present but inaccessible in the brain of animal models suffering from retrograde amnesia [48].

We posit memories are maintained in allocated cells. However, what prevents content retrieval during aging or AD pathology progression remains unknown. One likely candidate that curtails excitability of these cells is the APP metabolite amyloid-β [43]. Maintenance of collective synapses between memory allocated cells contributes to the recall of an excitable network [48]. For example, destruction of dendritic spines that hold excitatory synapses formed at the time of learning was sufficient to erase memory [25]. Therefore, the *connectivity* between task-activated cells stores memories. Such connections are lost in early stages of AD, as amyloid-β causes significant reorganization of the inhibitory-excitatory balance [14].

Discovering ways to maintain the connectivity of task-activated neurons for promoting memory retrieval in AD represents an important approach for the next generation of cognition therapeutics [48]. Several preclinical and clinical studies to enhance excitatory synapses have focused on modulating metabolism, proteostasis, barrier functions, synaptic transmission and inflammation [11]. However, a translational challenge is to find a selective target that would rule all of the others, alone or in combination with accessory therapies.

Hormones like cortisol organize body responses to internal and external demands with influential consequences on neuronal network activity [39]. Clinical studies suggest that patients with high levels of circulating cortisol have cognitive impairment and neurotoxicity [41], along with increased amyloid-β and tau pathology [24]. A polymorphism in 11β-hydroxysteroid dehydrogenase type 1 (11β-HSD1), the enzyme that generates cortisol directly in the brain is associated with an elevated risk for sporadic AD [17]. Chronic elevated levels of circulating cortisol are often comorbid with stress-related disorders (e.g., anxiety and depression), and also predicted to increase the risk of AD dementia in a prospective clinical study [19]. Accordingly, strategies to block the cortisol pathway have been a therapeutic focus. Full antagonists of the cortisol-binding glucocorticoid receptor (GR) reduced AD pathology and amnesia in mouse models [10, 36]. However, side effects are problematic, as oscillating cortisol signaling is required for multiple peripheral and brain functions including learning and memory [28]. Alternative strategies employing partial antagonists, inverse agonists of GR [44] or inhibitors of 11β-HSD1 [53] decreased neuropathology but displayed unwanted side effects on emotional memories in animal models. Several clinical trials are either ongoing (NCT03823404, NCT04601038) or stopped due to futility (NCT01137526).

GR is a potential drug target for AD, but it is ubiquitous, and its effect on large scale neuronal networks remains poorly understood [26]. For instance, the response to cortisol enhanced the excitability of memory allocated cells compared to the non-responders with respect to encoding and recall [35]. This suggests the cortisol signaling pathway is cued to behavioral experiences by influencing connectivity among cells allocated to specific tasks. To date, proof that GR mechanistically underlies this effect is lacking and requires further study.

We recently reported activity-dependent cortisol signaling through GR moderates Tau hyperphosphorylation and synaptic plasticity [9]. We previously identified GR is phosphorylated at distinct serine-proline consensus sites in the transactivation domain that are cortisol-dependent or neurotrophic-dependent [7]. Phosphorylation transforms the intrinsic disorder of the transactivation domain into order between dynamic transition states in the tertiary structure [37]. Surfaces adopted by GR conformation not only integrate the biochemical environment of the cell with cortisol-independent phosphorylation but also the external demands with cortisol-dependent phosphorylation. Mutations of cortisol-dependent sites alter docking of effectors (*e.g.* 14–3–3, FKBP5, HSP90, HDAC2 [8, 21, 33]) because phosphorylation decreases the energy requirement for folding the aminoacid chain directing protein–protein interactions, which is especially stable when the adjacent residue is a proline adopting cis/trans conformations [30]. Mutations of neurotrophic-dependent sites also decrease binding with effectors (*e.g.* CREB1, GRIP1, BRG1, 14–3–3 [6, 34]). The phosphorylation landscape on surfaces adopted by GR is expected to determine protein complex formation and signaling outcome in the neurodegenerating brain [23]. We found increased GR phosphorylation (p-GR) at neurotrophic-dependent (serines S134/S267) but not cortisol-dependent (serines S211/S226) sites in task-allocated neurons, and that neurotrophic-dependent p-GR was impaired in mice with reduced BDNF secretion [2]. Disruption of the BDNF-dependent p-GR sites in mice preserved cortisol-induced phosphorylation, and impaired synaptic plasticity in task-activated neurons [2]. Deletion of the activity-dependent GR pathway is distinct from a complete loss-of-function [26]. Importantly, the impact of disrupting neurotrophin-dependent p-GR on age-related cognitive decline and in relevant animal models of AD remains unknown. We tested how AD neuropathology seeded in mice by causal human variants would evolve if select p-GR pathways are deleted by knockin mutations known to impair adaptation to stress [2]. Herein, we tracked task-related memory performance and neuroimaging correlates during the early phase of AD pathology in the transgenic mouse expressing the AD variants APP^swe^ and PS1^dE9^ in the well-established line APP/PS1. Findings indicate that p-GR at neurotrophin-induced sites is reduced in postmortem AD brain, and mice genetically modified to disrupt neurotrophic-dependent p-GR are less efficient at maturing task-related synapses and memory performance without impacting amyloid-β accumulation. Collectively, these data suggest that *increasing* p-GR at the sites induced by neurotrophic signaling could improve cognition in AD subjects without impacting cortisol-activating sites, presenting a novel opportunity for therapeutic intervention.

## Materials and methods

### Humans

Frozen tissues of the prefrontal cortex [Broadman areas 9–10] (n = 79) subjects with age at death ranging from 29 to 98 years (yr), and postmortem interval (PMI) ≤ 35 h (h) were obtained from the following brain banks: Rush Religious Orders Study (RROS), Center for Neurodegenerative Disease Research, University of Pennsylvania School of Medicine, Harvard Brain Tissue Resource Center and the Emory Center for Neurodegenerative Disease, Emory University School of Medicine. A total of n = 21 control subjects (12 M/9F) were clinically examined and diagnosed with no cognitive impairment or insufficient to meet criteria for dementia (age: 69.8 ± 3.6 yr, PMI: 13 ± 1.8 h, Braak stage: 1.3 ± 0.23). Neurodegenerative disorder cases include n = 40 AD (14 M/26F, age: 78.6 ± 1.8 yr, PMI: 11.3 ± 0.9 h, Braak stage: 5.6 ± 0.07) and n = 18 Parkinson’s disease (PD; 12 M/6F, age: 75.4 ± 2.13 yr, PMI: 8.9 ± 1 h, Braak stage: 1 ± 0.17). Cognition was assessed within the year prior death using the Mini-Mental State Exam (MMSE). Scores are 29.2 ± 0.35 for controls, 12.2 ± 1.3 for AD and 26 ± 1 for PD. Exclusion criteria for AD cases included argyrophilic grain disease, frontotemporal dementia, Lewy body disease, mixed dementias, PD, and stroke. Neuropathology was determined by a board certified neuropathologist blinded to the clinical diagnosis. Tissue samples were processed as previously described [22] for Western blotting using the following antibodies: p-GR[S267], p-GR[S134], p-GR[S211], p-GR[S226] (1:1000, all made by M. Garabedian, New York University Grossman School of Medicine (NYUGSOM; NY, NY, USA [7]), total GR (1:400, P20, Santa Cruz Biotechnology, Santa Cruz, CA, USA), APP (1 μg/ml, PA1-84,165) and anti-oligomer A11 (1 μg/ml, ThermoFisher, Waltham, MA, USA), Tau-1 (1:1000, Sigma-Aldrich MAB3420, USA), PHF1[p-Tau S396/S404] (1:500, a gift of P. Davies, Long Island Jewish Medical Center, Northwell Health, New Hyde Park, NY, USA), GAPDH (1:1000, Meridian Life Sciences, Menphis, TE, USA), FKBP5 (1:1000, Abcam ab2901, Paris, France), TrkB (1:1000, 610,101, BD Biosciences, USA), p-TrkB[Y816] (1:1000, a gift of M.V. Chao, NYUGSOM), BDNF (1:400, N20, Santa Cruz Biotechnology) and HSP90 (1:1000, 610,418, BD Biosciences). Total protein levels were determined with bicinchoninic acid (BioRad, Courtaboeuf, Les Ulis, France) against known concentrations of bovine serum albumin (BSA). Fifty μg of proteins were loaded in each well of 4–12% acrylamide/bis-acrylamide gels ran in denaturing conditions then transferred onto PVDF membranes for immunodetection. HRP activity conjugated to secondary antibodies was revealed with ECL substrate (Amersham, Bethesda, MD, USA). Images were subtracted of background with ImageJ and optical densities of bands normalized to GAPDH and GR.

### Animals

Transgenic lines Thy1-YFP (*B6.Cg-Tg(Thy1-YFP)HJrs/J,* APP/PS1 (*B6C3-Tg(APPswe,PSEN1dE9)85Dbo/Mmjax)* are from Jackson labs (Bar Harbor, ME, USA), and NR3C1 knockin mutant Ser134Ala/Ser267Ala (*B6.Tg(Nr3c1*^*tm2/Jean*^*)/J)* was previously described [2]). Mice were housed in groups (2–4/cage) with cotton swabs and igloos for nesting, under a 12 h light/dark cycle (on 7 AM, off 7 PM), at 22–24 °C, 50 ± 5% humidity, and *ad-libitum* food and water. All efforts were made to minimize animal suffering and reduce their number in each experiment. The starting number of mice used included (n = 19 *Nr3c1*^+/+^-thy1-YFP, n = 16 *Nr3c1*^ki/ki^-thy1-YFP, n = 26 *Nr3c1*^+/+^-thy1-YFP-APP/PS1 and n = 19 *Nr3c1*^ki/ki^-thy1-YFP;APP/PS1), which decreased in the later age points due to mortality compounded by genotypes and anesthesia during imaging. Mice were acclimated at least 1 h in testing rooms before behavior. Behavior testing was always done in mornings (8 A.M.–12 P.M.) except for the rotarod training (7 P.M.). Equipment was cleaned thoroughly with 30% ethanol between trials. Longitudinal data were collected at 3, 6 and 9 months in different testing rooms by different experimentalists blinded to the age and genotype of the animals. We used both males and females for behavioral characterization of the *Nr3c1*^ki^ line. We used males only for all multiparametric experiments in multi-transgenic animals.

### Open field

Mice positioned in the center freely explored an arena (50 cm × 50 cm, dim light ~ 50 lux) for 10 min filmed with a webcam. Total distance traveled and time spent in the center (29 cm × 29 cm) were determined with EzTrack (available on Github).

### Elevated plus maze

Mice positioned in the center freely explored the arms (50 cm × 20 cm elevated 50 cm above floor, dim light ~ 20 lux) for 5 min filmed with a webcam. The number of entries and time spent in each arm were determined manually.

### Rotarod training

Mice were habituated on the non-accelerating rotarod (2 rpm, 1 min followed by 30 s rest, repeated 15 trials) for 2 consecutive days before 2 training sessions each of 15 trials on the accelerating rod (from 2 to 80 rpm reached in 2 min with 1 min rest inter-trial) for 2 consecutive days in the evenings (~ 5 lux). Recall was performed 10 days later for 1 session on the accelerating rod as before.

### Novel object recognition

Mice positioned in the center freely explored a L-shaped arena (30 cm × 10 cm, dim light ~ 50 lux) for 10 min filmed with a webcam on day 1 for habituation, with identical objects on each side on day 2, and with one previous (Lego blocks) and one novel object (falcon tube) on each side on day 3. Time spent exploring each side on day 1 and touching the objects on day 2 and 3 were determined manually. Object preference was calculated as ratio of time spent with each object; object memory was calculated as index = (novel − known)/(novel + known).

### Three-chamber test

Mice positioned in the center freely explored an arena (60 cm × 41 cm divided in 3 equal chambers with 2 doors in the middle, dim light ~ 50 lux) with empty prisons on each side for 10 min filmed with a webcam on day 1, on day 2 with a same-sex juvenile on one side to determine preference, and on day 3 with the previous juvenile on same side and one novel on the other to determine memory. Time spent touching the empty prisons or the juveniles were determined manually. Social preference was calculated as ratio of time spent with the juvenile over the empty prison; social memory calculated as index = (novel − known)/(novel + known).

### Barnes maze

Mice positioned in the center explored an arena (92 cm diameter with 20 holes 5 cm each equally spaced, one of which has the hidden escape box elevated 105 cm above floor, bright light ~ 100 lux) for the time necessary to guide the mouse in the correct hole to spend 2 min in the escape box for habituation on day 1. During acquisition on day 2, mice are given 3 min to find freely the hidden box and reside for 1 min. Mice are place back in homecage for 15 min before next trials (repeat 3 times/day for 5 days). Probe trials were conducted for 90 s on day 6 and 14 in which the hidden box is removed to test for short and long term memory. Number of pokes (errors) in each hole and latency to reach the target hole was measured manually.

### Y-maze

Mice positioned in the center freely explored the arms (60 cm × 15 cm) for 5 min filmed with a webcam. We counted manually the number of alternations between consecutive arms.

### Thinned skull 2-photon microscopy

Mice were anesthetized with a mix of 0.075 mg/g ketamine and 0.01 mg/g xylazine and lidocaine sprayed atop the skull prior surgery. Skull bone was thinned to transparency using disposable ophthalmic surgical blades (Surgistar, Vista, CA, USA). The scalp is sutured and topped with antibiotic cream to avoid infection between imaging sessions. A detailed map of the pial vasculature and dendritic territories were taken for subsequent relocation as previously described [5]. Only males were used because YFP expression is too bright and diffuse in females interfering with the detection of quantifiable isolated dendrites and spines specifically in aged females.

### Open skull 2-photon microscopy

A 3–4 mm craniotomy was prepared over the transcranial imaging zone and the underlying dura was removed and kept in an aqueous environment of HEPES-buffered artificial cerebrospinal fluid (ACSF in mM 120 NaCl, 3.5 KCl, 0.4 KH2PO4, 15 glucose, 1.2 CaCl2, 5 NaHCO3, 1.2 Na2SO4, 20 HEPES, pH = 7.4). The cortex was covered by a thin layer of low-melting agarose 0.8% in ACSF to avoid heartbeat motion artifacts as previously described [2]. Hamilton syringe with a glass pipette were used to deliver 1–2(-nitrophenyl)ethyl(S)AMPA at 5 mM or glutamate at 100 mM (Bio-Techne, France) diluted in ACSF through the agarose bed as previously described [3]. Photolyse parameters were tuned to 720 nm, 0.7 mW for 5 s, and directed in motor cortex specifically at the head of new spines formed after the rotarod training. Images were taken for up to 15 min (n = 19 *Nr3c1*^+/+^-thy1-YFP, n = 33 *Nr3c1*^ki/ki^-thy1-YFP). Control spines did not receive laser stimulation (n = 13 *Nr3c1*^+/+^-thy1-YFP, n = 15 *Nr3c1*^ki/ki^-thy1-YFP). Spine enlargement was calculated as the % change of brightness in the head defined as region of interest using ImageJ [2].

### Image acquisition

Mice (n = 9 *Nr3c1*^+/+^-APP/PS1-thy1-YFP, 8 *Nr3c1*^ki/ki^-APP/PS1-thy1-YFP) were injected i.p. with 10 mg/kg Methoxy-Xo4 (Bio-Techne) 48 h prior to imaging at 3, 6 and 9 months of age. Just before acquisition, mice were also injected i.v. with 50 μl dextran 70 Kda (25 mg/ml conjugated with Texas red or FITC, Sigma-Aldrich, St. Louis, MO, USA). Hydrazide-AlexA633 (1 mg/kg, ThermoFisher Scientific) was injected i.v. 24 h prior to imaging to mark arterioles. Images were acquired in the somatosensory cortex of deeply anesthetized mice with a FVMPE RS two-photon microscope (Olympus, Hamburg, Germany) equipped with a 25X, numerical aperture 1.05 water-immersion objective (XLPLN25XWMP2, Olympus) and an InSight X3 femtosecond-pulsed infrared laser (Spectra-Physics, Evry, France) for optimal fluorescence excitation and emission separation. Excitation was at 750 nm for Methoxy-Xo4, 780 nm for FITC, 960 nm for YFP and 1040 nm for Texas Red. Images were taken with a digital zoom of 7.2 at each image session using 0.75 μm step with a scanning dwell time of 2.55 μsec per pixel. Laser power was adjusted with the depth but kept below 30 mW. Each scan stacks consists of images at 512 × 512 pixels resolution. Time-lapse acquisition was done in a smaller field of view with galvanometric scanning mode and conventional raster scanning for blood flow measurements based on the line scanning method [4]. Plasma is fluorescent unlike blood cells that do not uptake dextran dyes permitting identification of their circulation. Fluorescence excitation was delivered by a Lambda LS xenon arc lamp (300 W; Sutter Instruments, Novato, CA, USA) fitted with a fast-rotating filter wheel (27 ms lag) and linked to the stereomicroscope with an optical fiber and 20 × objective. Fluorescence emission was captured with a sCmos camera (C11440 Orca-Flash 4.0, Hamamatsu Photonics, Japan) capturing images at 200 Hz that allowed speed limit up to 9 mm/s which is sufficient for capturing flow in arterioles ~ 10 μm diameter. In a separate experiment, mice (n = 4 *Nr3c1*^+/+^-APP/PS1-thy1-YFP, n = 4 *Nr3c1*^ki/ki^-APP/PS1-thy1-YFP not trained and n = 6 *Nr3c1*^+/+^-APP/PS1-thy1-YFP, n = 6 *Nr3c1*^ki/ki^-APP/PS1-thy1-YFP trained) were injected i.p. with corticosterone (15 mg/kg, Sigma-Aldrich) 12 h after the last rotarod training, and immediately after the first imaging of the time-lapse session.

### Image analysis

The field of view (200 × 200 × 150 μm) in consecutive images was realigned with RegStack plugin and distances between nearest spines along dendrites and from amyloid plaques measured with ImageJ. Regions of interest were drawn to measure the surface of amyloid deposits. The numbers of blood cells were counted in the amyloid-covered vessels in 0.15 mm^3^ of field of view and normalized to 1 mm^3^. The number of axonal dystrophies and dendritic spines were expressed as densities. Dextran-Texas Red filled microcirculation but did not penetrate blood cells permitting identification of their circulation as previously described [4]. The change of flow between imaging sessions was determined only in amyloid-covered vessels. Two or more additions (or eliminations) of spines ≤ 5 μm along a dendrite define a dynamic cluster of formation (or elimination) as previously described [18]. All clear headed-protrusions emanating laterally from the dendritic shaft were counted. Approximately 200 dendritic spines from at least 10–20 dendritic segments were counted per conditions throughout the imaging sessions and averaged per animal. The presence, loss and gain of spines were counted between sessions for each segment and plotted as a function of distance to the nearest spine and amyloid plaque. We verified the distance perimeter from a plaque in 3D. Distance measurement between spines was set at the base of the neck to the base of the next spine following the trace of the dendritic shaft. The proportion of clustered formation (or elimination) equals the number of spines in clusters divided by the total number of new spines added (or eliminated) between imaging sessions.

Simulations of the distance between the nearest spine added (or eliminated) were performed to test if the observed distance is different from chance. For this, one dynamic spine was kept in its fixed position while the other dynamic spines were permutated randomly. This operation was repeated as many times as the number of dynamic spines. For each permutation, one spine of a cluster was randomly re-assigned to all possible spine positions on that dendritic segment keeping the other spine in its fixed observed position. Matlab was used to measure the distance between the clustered spines for each permutation and repeated the process 30,000 times to calculate a 99.9% confidence interval for the probability of clustering as previously described [18]. Averaged values yielded a random distribution of any possible spine clusters in defined dendritic territories from which we calculated the Gaussian best-fit value (Mean ± SEM) to which we compared the observed value. Matlab was also used to simulate the restoration of lost spines at any spine position in the dendrite. We measured the distance between a lost spine and the restored one for each permutation (restoration if < 2 μm, de novo addition if > 2 μm) and repeated the process 10,000 times to calculate a 99.9% confidence interval for the probability of restored lost spines as previously described [40].

### Electrophysiology

On the next day of the last training session, coronal slices ≈400 μm of motor cortex were cut with a vibratome and transferred to a temperature controlled (34 ± 1 °C) chamber perfused with oxygenated ACSF (in mM: 127,25 NaCl, 1,75 KCl, 1.25 KH_2_PO_4_, 1 MgCl_2_, 2 CaCl_2_, 26 NaHCO_3_, 10 glucose) at a rate of 1 mL/min. Stimulation electrodes were positioned in layer 2/3 ≈500 μm away from recording electrodes. Field potentials (FP) were evoked by stimulation of 0.2 ms at 0.03 Hz. Amplitudes were recorded using single stimuli applied every 30 s for at least 30-min to reach stable baseline. High-frequency stimulation (HFS) consisted in 10 trains of 5 Hz stimuli, each composed of 4 (200 ms) pulses at 100 Hz, repeated 5 times every 10 s. Low-frequency stimulation (LFS) consisted in 2 Hz stimulus for 15 min. Stimulus intensity eliciting 50% of the maximum amplitude was used for all measurements to assess the physiological range of saturation upon repeated protocols as described [46]. The GABA_A_ antagonist Bicuculline methiodide (3.5 mM) was applied at the end of recordings to establish the health of the slices and washout with ASCF.

## Immunohistology

Brains were harvested in mornings (8 AM- 12 PM) following transcardial perfusion with phosphate buffered saline (PBS) followed with 4% paraformaldehyde, and post-fixed for 24 h at 4 °C. Free-floating coronal sections of 40 μm obtained with a vibratome, were rinsed in PBS then blocked in 5% normal goat (or donkey) serum, PBS, 0.1% triton X-100 for 2 h at 25 °C. Primary antibodies, GFP (1:3000, ab13970, Abcam), Iba1 (1:1000, ab5076, Abcam), GFAP (1:1000, Merck, Darmstadt, Germany), p-GR[Ser267], p-GR[Ser246] (1:1000, M. Garabedian, NYUGSOM) were incubated for 2 days at 4 °C and secondary antibodies (1:2000, ThermoFisher Scientific) for 2 h at 25 °C. Sections were washed in PBS, 0.1% Triton and mounted in Fluoromount (Sigma Aldrich). Fluorescence images were taken with an apotome microscope (Carl Zeiss) equipped with water immersion objectives or with LSM780 laser-scanning confocal microscope (Carl Zeiss, Iena, Germany) equipped with Plan-Neofluor NA1.3 oil-immersion objectives.

### Preparation of Aβ42 oligomers

Aβ42 peptides (ERI Amyloid Laboratory LLC, Oxford, CT, USA) were maintained in a monomeric state using the protocol described in [50]. Briefly, Aβ42 peptides were dissolved in a 6.8 M guanidine thiocyanate solution (Sigma-Aldrich) at a concentration of 8.5 mg/ml. The solution was then sonicated for 5 min at 52 °C, and diluted with ultrapure water to reach a final concentration of 5 mg/ml of Aβ42 peptides and 4 M of guanidine thiocyanate. The solution was centrifuged at 10,000 g for 6 min at 4 °C. The supernatant was filtered and then injected into a Superdex 75 Increase 10/300GL column (GE Healthcare Life Science, Chicago, IL, USA) previously equilibrated with 10 mM sodium phosphate buffer pH 7.4. The peak attributed to monomeric Aβ42 was collected and the concentration was determined from absorbance. Stock solution was diluted to 90 μM in 10 mM sodium phosphate, pH 7.4 and left to aggregate in a low-binding Eppendorf tube. The tube was arranged vertically and incubated at 25 °C under quiescent state. Amyloid formation was monitored by thioflavin T (ThT) binding. To do so, aliquots were withdrawn at different time points and ThT fluorescence was measured (λex = 445 nm and λem = 485 nm) in a Fluoroskan plate reader (ThermoFisher Scientific). Once Aβ aggregation reached the early stages of the growth phase, the sample was flash frozen and maintained at − 20 °C. Oligomers were used as positive control in a dot blot to detect soluble oligomeric amyloids in the somatosensory cortex (N = 4/group). Optic densities were measured with Image J (median ± SEM 59 ± 16 and 62 ± 57 between *Nr3c1*^+/+^-APP/PS1 and *Nr3c1*^ki/ki^-APP/PS1 mice, Mann–Whitney test *P* = 0.8).

### Intracerebroventricular (i.c.v.) injection

We injected mice (n = 9 *Nr3c1*^+/+^ and n = 8 *Nr3c1*^ki/ki^) with 1 μM (2.2 nmol) of oligomers i.c.v. via a stereotaxic frame (AP + 0.22 cm, ML ± 1 cm, DV -2.5 cm) connected to 10 μl Hamilton syringe controlled by a microinjector pump (micro4, World Precision Instruments, USA) at a rate of 0.5 μl/min. The needle was left in place for 5 min post-injection prior to retraction. The wound was disinfected, sutured and animals left to recover for a week before behavioral testing. For dendritic spine imaging, we injected *Nr3c1*^ki/ki^-thy1-YFP mice with oligomers (n = 7/group), controls received vehicle (n = 7/group). Mice were prior trained on the rotarod for 2 days to promote spine survival in the motor cortex. Images were acquired in the motor cortex before and after the training to identify pre-existing and newly formed spines, and 7 days after oligomers injection to identify their survival and dynamics.

### Blood sampling

Five μl of blood was collected from the tail vein in the morning and in the evening of the same mice (n = 5/group) before and after i.c.v. injection of oligomers. Corticosterone blood levels were measured by ELISA (Enzo Life Sciences, Villeurbanne, France) per manufacturer instructions.

### Statistics

All investigators were blind to experimental status during the acquisition and analyses of data. Normal distribution of data was tested with the Shapiro–Wilk test. Pairwise comparisons were done with two-tailed t-test (Kruskal–Wallis or Mann–Whitney). Correlations were determined with the Pearson coefficient. Multi-parametric comparisons were done with ANOVA followed by post-hoc corrections with appropriate tests (e.g. Sidak, Tukey) using Prism 8.0 (GraphPad, San Diego, CA, USA). Estimates of sample size were calculated using G-power analysis software based on previous studies and preliminary data, and were powered to detect moderate, biologically meaningful effect sizes.

## Results

### GR phosphorylation in Alzheimer’s disease

**Fig. 1:**
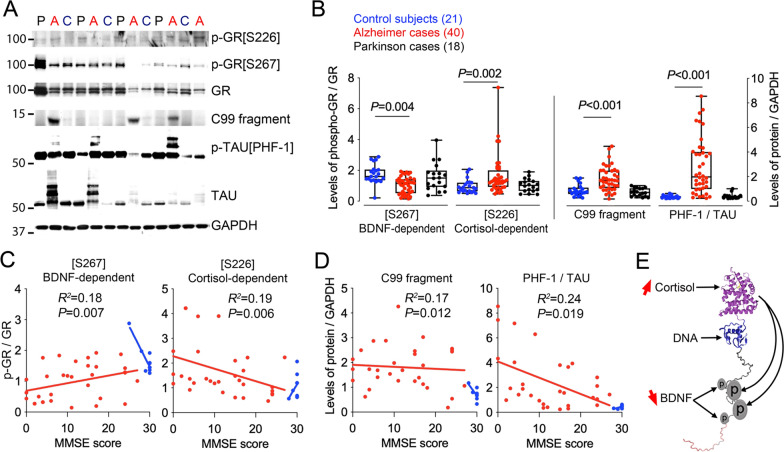
GR phosphorylation is altered in Alzheimer Disease. **a** Western blot of proteins in the prefrontal cortex (C, controls, A, Alzheimer, P, Parkinson). **b** Optical densities for the indicated protein markers. Quartiles and median of dataset from Min-to-Max. Significant differences with Mann–Whitney test between 21 C and 40 A is not observed between 21 C and 18 P (*P* = 0.8 for S267, *P* = 0.2 for S226, *P* = 0.4 for C99 and *P* = 0.9 for PHF-1). **c** Correlation between scores on the MMSE performed in a subset of patients within a year prior death (10 C and 29 A) and p-GR levels is positive (Pearson r = 0.35) for the site [S267] and negative (Pearson r = − 0.39) for the site [S226]. **d** Correlation between MMSE scores and the levels of C99 (Pearson r = − 0.07) and PHF-1 (Pearson r = − 0.46). **e** Cortisol receptor’s structure (modified from [37]) is highly disordered in its transactivation domain that is phosphorylated at multiple sites via distinct pathways altered in AD (red arrows)

We performed western blot analysis of lysates from human prefrontal cortex to determine relative expression levels of markers in the brain of AD subjects compared to non-demented age-matched controls. Antibodies to C99 fragment of APP and p-Tau indicated elevated levels in AD cases compared to controls, serving as surrogates of pathological proteases and kinases activities that confirmed diagnosis (Fig. 1a, b). The levels of p-GR were normalized to the levels of total GR and to the loading control GAPDH. We observed a significant decrease in GR phosphorylation at the BDNF-responsive site p-GR[S267] in AD (− 40%, *P* = 0.004). In contrast, subjects diagnosed with an unrelated neuropathology (Parkinson’s Disease) showed no differences in p-GR at this same site (+ 4%, *P* = 0.8), indicating disease specificity. Data were also stratified according to PMI, gender, age (Additional file 1: Fig. S1), and cognitive performance (MMSE) obtained within the year of death (Fig. 1c, d). The levels of p-GR[S267] *positively* correlated with the MMSE score (*P* = 0.007). This contrasted with the *negative* correlation between MMSE score and p-GR levels at the cortisol-dependent site p-GR[S226] (*P* = 0.006) that were increased in AD subjects compared to controls (+ 72%). Sex and age were not associated with p-GR, except in the AD group for p-GR at the S226 site (Additional file 1: Fig. S1a, b). We confirmed these observations in AD at an additional BDNF-dependent site p-GR[S134] (− 29%, *P* = 0.03) and another cortisol-dependent site p-GR[S211] (+ 48%, *P* = 0.05, Additional file 1: Fig. S2).

In addition, the levels of BDNF and activated phosphorylated TrkB (p-TrkB) were decreased in AD cases (− 33%, *P* = 0.001 and − 27.4%, *P* = 0.03, respectively) contrasting the levels of total TrkB and GR, which did not differ (Additional file 1: Fig. S3). Chaperones of GR (e.g. FK506 binding protein 5 (FKBP5) and heat-shock protein 90 (HSP90), were also found to have significantly reduced expression levels in AD compared to controls (FKBP5, − 34%, *P* = 0.002 and HSP90, − 51%, *P* < 0.0001, Additional file 1: Fig. S3). Neither PMI nor aging associated with differential expression between cohorts. Taken together, GR phosphorylation at the BDNF-dependent sites is decreased, whereas the cortisol-induced sites are increased in AD.

### Mice with impaired p-GR status exacerbate AD-like progression in the APP/PS1 model

**Fig. 2:**
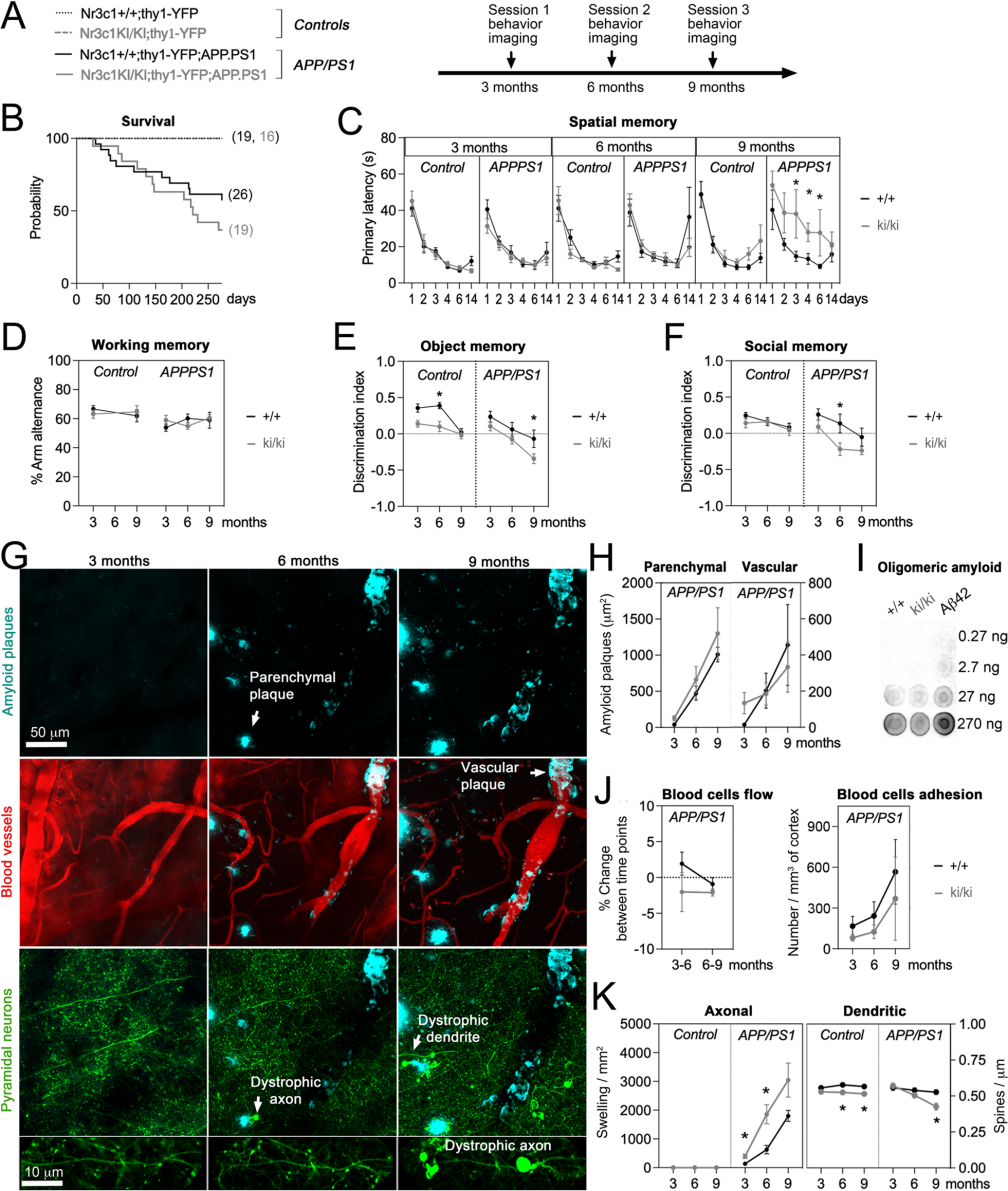
Lack of p-GR sites causes neurodystrophies, learning and memory deficits in the APP/PS1 model without impacting amyloid deposition and cerebrovascular pathology. **a** Experimental timeline in triple transgenic mice. **b** Survival over 9 months: effects of APP/PS1 (Chi2 = 26.9 df = 3, *P* < 0.001) and *Nr3c1*^ki/ki^ (Chi2 = 1.3 df = 1, *P* = 0.25). **c** Primary latency to find the hidden platform in the Barnes maze during training (days 1–4) and recalls (days 6 and 14). Means ± SEM of N_(3,6,9 months)_ = 10,9,9 *Nr3c1*^+/+^, 9,9,9 *Nr3c1*^ki/ki^, 12,8,8 *Nr3c1*^+/+^-APP/PS1, 14,10,10 *Nr3c1*^ki/ki^-APP/PS1 mice. Two-way ANOVA: effect of genotype *F*_7,68_ = 4.7, *P* = 0.0002; effect of time *F*_5,332_ = 63.3, *P* < 0.0001 post-hoc Tukey test *P < 0.05. **d** Percentage of alternance between arms of a Y-maze. Means ± SEM of N_(3,9 months)_ = 12,9 *Nr3c1*^+/+^, 12,9 *Nr3c1*^ki/ki^ and N_(3,6,9 months)_ = 7,7,6 *Nr3c1*^+/+^-APP/PS1, 13,13,5 *Nr3c1*^ki/ki^-APP/PS1 mice. Two-way ANOVA analyses show no effect of genotype or aging *P* > 0.05. **e** Time exploring the new object over the old one presented 24 h earlier in the novel object recognition test. Means ± SEM expressed as ratio index in N_(3,6,9 months)_ = 15,15,13 *Nr3c1*^+/+^, 16,16,11 *Nr3c1*^ki/ki^, 10,8,5 *Nr3c1*^+/+^-APP/PS1, 16,9,5 *Nr3c1*^ki/ki^-APP/PS1 mice. Three-way ANOVA: effect of APP/PS1 *F*_1,127_ = 18.3; effect of *Nr3c1*^ki/ki^ *F*_1,127_ = 20.6; effect of aging *F*_2,127_ = 16.9, *P* < 0.0001 post-hoc Tukey test *p < 0.05. **f** Time exploring the new mouse over the old one presented 24 h earlier in the 3-chamber test. Means ± SEM expressed as ratio index in N_(3,6,9 months)_ = 12,10,9 *Nr3c1*^+/+^, 12,10,9 *Nr3c1*^ki/ki^, 10,8,6 *Nr3c1*^+/+^-APP/PS1, 16,11,5 *Nr3c1*^ki/ki^-APP/PS1 mice. Three-way ANOVA: effect of APP/PS1 *F*_1,106_ = 7; effect of *Nr3c1*^ki/ki^ *F*_1,1106_ = 11.9; effect of aging *F*_2,106_ = 6, *P* < 0.005 post-hoc Tukey test *p < 0.05. **g** Repeat images of a cortical volume in somatosensory cortex of *Nr3c1*^+*/*+^ mouse at 3, 6 and 9 MO to track in 3D the amyloid plaques (methoxy-XO4), blood vessels (75KDa dextran-AlexA594) and pyramidal neurons of layer 5 (thy1-YFP). **h** Surface of parenchyma and vascular amyloid plaques. Means ± SEM of N_(3,6,9 months)_ = 11,7,5 *Nr3c1*^+/+^-APP/PS1, 13,11,7 *Nr3c1*^ki/ki^-APP/PS1 mice. Not determined (n.d). Two-way ANOVA: effect of genotype *F*_1,22_ = 1.9, *P* = 0.18; effect of aging *F*_2,26_ = 34.3, *P* < 0.0001. **i** Oligomeric amyloid detected with A11 antibody in somatosensory cortex (5 μg and 1/5 serial dilutions) of *Nr3c1*^+/+^-APP/PS1 and *Nr3c1*^ki/ki^-APP/PS1 mice (8 months old) against a range of diluted Aβ42 oligomers prepared in vitro. **j** Flow and adhesion of blood cells in amyloid-covered vessels. Means ± SEM of N_(3,6,9 months)_ = 6,6,6 *Nr3c1*^+/+^-APP/PS1, 8,8,6 *Nr3c1*^ki/ki^-APP/PS1 mice. Two-way ANOVA: effect of time on adhesion *F*_1,20_ = 5.2, *P* = 0.015; no effect on flow. **k** Number of dystrophic axons and dendrites of pyramidal neurons in somatosensory cortex. Means ± SEM of N_(3,6,9 months)_ = 3,3,3 *Nr3c1*^+/+^, 3,3,3 *Nr3c1*^ki/ki^, 9,5,3 *Nr3c1*^+/+^-APP/PS1, 12,8,4 *Nr3c1*^ki/ki^-APP/PS1 mice. Three-way ANOVA: effect of APP/PS1 on axons *F*_1,23_ = 52.9, *P* < 0.0001 and dendrites *F*_1,19_ = 2.7; effect of *Nr3c1*.^ki/ki^ on axons *F*_1,23_ = 6.1 and dendrites *F*_1,14_ = 9.1, *P* < 0.01; effect of aging on axons *F*_2,24_ = 26 and dendrites *F*_2,28_ = 26.2, *P* < 0.0001 post-hoc Tukey test *P < 0.05

To test if alterations in BDNF-dependent p-GR affect the onset and/or progression of AD-like neuropathology and cognitive decline, we crossed mice carrying a knockin allele lacking the conserved BDNF-responding phosphorylation sites orthologous to S134 and S267 in GR (*Nr3c1* gene) that we previously described [2] with mice expressing the AD variants APP^swe^ and PS1^dE9^ in the well-established line APP/PS1 [45]. In this model, disease becomes symptomatic between 6–9 months of age (MO) [36], which prompted us to investigate endophenotypes at 3 time points to track progression as a function of GR mutations (Fig. 2a). GR phosphorylation at S267 is brain-specific (Additional file 1: Fig. S4) and predominantly neuronal (Additional file 1: Fig. S5). To visualize excitatory neurons with transcranial microscopy, we used the thy1-YFP line [38] and upon crossing them with the two other lines in a triple transgenic model. To visualize non-neuronal pathology, we also injected fluorescent tracers of blood vessels and amyloid.

We observed APP/PS1 mice carrying the NR3C1-S134A/S267A allele (referred to as ki/ki) were more likely to die (~ 27%) than APP/PS1 mice carrying the *Nr3c1*^+/+^ allele, even though homozygosity is not lethal per se (Fig. 2b). *Nr3c1*^ki/ki^ did not behave differently from *Nr3c1*^+/+^ mice in terms of locomotion, anxiety, working memory, social and non-social memory (Additional file 1: Fig. S6). Sex differences were not found at 3 MO using a battery of behavioral tests. However, several types of memory were altered in *Nr3c1*^ki/ki^-APP/PS1 compared to *Nr3c1*^+/+^ controls across aging. Specifically, spatial learning and memory monitored in a Barnes maze showed comparable performance at 3 and 6 MO between *Nr3c1*^ki/ki^-APP/PS1 and *Nr3c1*^+/+^-APP/PS1 genotypes. However, at 9 MO, the latency to find the correct choice in the learning and memory phases of the test was poorer (+ 100%) in *Nr3c1*^ki/ki^-APP/PS1 mice compared to *Nr3c1*^+/+^-APP/PS1 littermates (*P* < 0.05, Fig. 2c). This contrasted with the lack of an effect in the Y-maze, another spatial task that relies on short-term working memory to navigate the maze (Fig. 2d). The use of the novel object recognition task and the 3-chamber test demonstrated the interaction of APP/PS1 and *Nr3c1*^ki/ki^ genotypes altered both non-social and social memory (+ 100%). This effect was revealed with increasing age, emerging late in the non-social task (9 MO, *P* < 0.05, Fig. 2e) and early in the social test (6 MO, *P* < 0.05, Fig. 2f). Taken together, *Nr3c1*^ki/ki^ mice exhibited poorer cognitive functions than *Nr3c1*^+/+^ in the APP/PS1 model of AD.

Although *Nr3c1*^ki/ki^-APP/PS1 mice displayed accelerated age-related cognitive deficits, other indicators of AD-like pathology by neuroimaging were unchanged (Fig. 2g and Additional file 1: Fig. S7). Specifically, amyloid deposition progressed at similar rates in the parenchyma as on blood vessels in somatosensory cortex (Fig. 2h). Levels of oligomeric amyloid were not different between genotypes at 8 MO (Fig. 2i). Likewise, blood flow and cell adhesion to vessel walls progressed independent of genotype (Fig. 2j, Additional file 1: Fig. S8). In contrast, neuronal dystrophies progressed differentially between genotypes. Axon swelling and dendritic spine loss were more prominent in *Nr3c1*^ki/ki^-APP/PS1 than disease controls (+ 69% and − 19% respectively, *P* < 0.05, Fig. 2k). *Nr3c1*^ki/ki^-APP/PS1 mice displayed neuronal defects and cognitive impairment earlier than controls while non-neuronal indicators of pathology were unaffected.

### Synaptic pathology consistent with memory deficits resulting from altered GR signaling

**Fig. 3:**
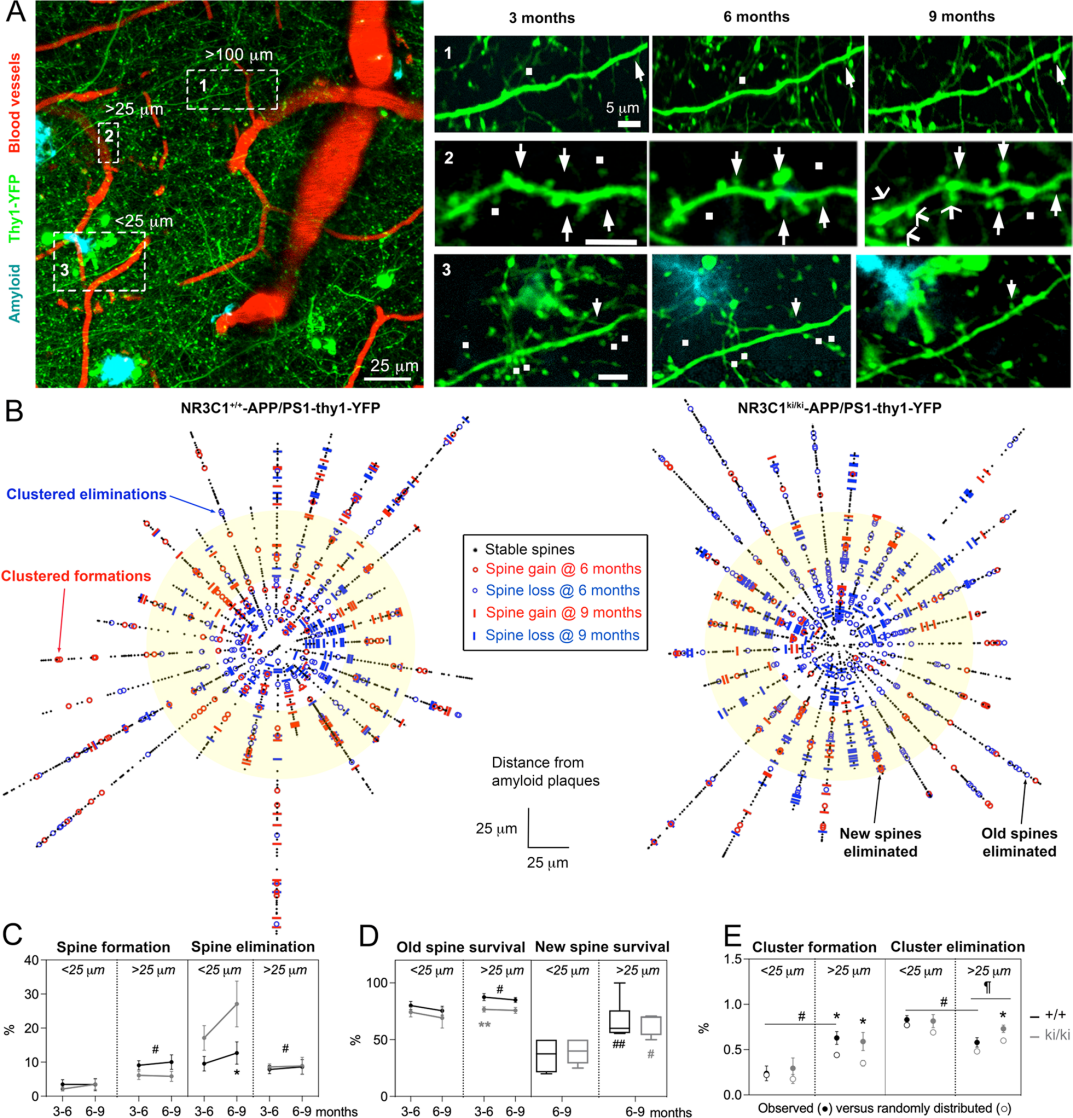
GR phosphorylation influences dendritic spines plasticity as a function of amyloidogenesis. **a** Field of view imaged 3 times in triple transgenic mice. **b** Remodeling of dendritic spines between time points as a function of distance to the nearest amyloid plaque centered in the dendrospinogram. Yellow circle shows the zone at 25-to-60 μm from the amyloid plaque. **c** Quantitative dynamics proximal and distal of amyloid plaques. Means ± SEM of N_(3,6,9 months)_ = 9,9,7 *Nr3c1*^+/+^-APP/PS1 and 10,10,5 *Nr3c1*^ki/ki^-APP/PS1 mice. Three-way ANOVA: effect of genotype on formation *F*_1,34_ = 3.95, *P* = 0.05 and elimination *F*_1,17_ = 6.67, *P* = 0.08; effect of distance on formation *F*_1,34_ = 13.2, *P* = 0.0009 and elimination *F*_1,17_ = 13.4, *P* = 0.002; effect of aging on formation *F*_1,21_ = 0.12, *P* = 0.7 and elimination *F*_1,17_ = 2.9, *P* = 0.1 post-hoc Tukey test comparing distances ^#^*P* < 0.05. **d** Survival of dendritic spines proximal and distal of amyloid plaques. Old spines (means ± SEM) of N_(3,6,9 months)_ = 10,10,8 *Nr3c1*^+/+^-APP/PS1; 10,10,5 *Nr3c1*^ki/ki^-APP/PS1 mice. Three-way ANOVA: effect of APP/PS1 *F*_1,3_ = 5.1, *P* = 0.1; effect of distance *F*_1,18_ = 7.7, *P* = 0.01 post-hoc Tukey test comparing distances ^#^*P* < 0.05 and genotypes *P < 0.05. New spines (Quartiles and median of dataset from Min-to-Max) of N_(<25,>25μm)_ = 4,7 *Nr3c1*^+/+^-APP/PS1 and 5,5 *Nr3c1*^ki/ki^-APP/PS1 mice. Two-way ANOVA: effect of genotype *F*_1,17_ = 0.001; effect of distance *F*_1,17_ = 21.3, *P* = 0.0002 post-hoc Tukey test comparing distances ^#^*P* = 0.02, ^##^*P* = 0.003. **e** Spine clustering is greater than chance level distal of amyloid plaques. Mean ± SEM of N_(+/+; APP/PS1, ki/ki;APP/PS1)_ = 6, 8, 8 mice. Two-tailed unpaired t-test comparing observed and simulated *P < 0.05, distal and proximal ^#^*P* < 0.001 or genotypes.^¶^*P* = 0.04

In depth analysis of dendritic spines dynamics in excitatory neurons provided clues about the relationship between altered p-GR status and cognitive impairment. Spine formation, elimination and survival between time points were examined in 3 zones of the imaging field: proximal (< 25 μm), intermediate (25–60 μm) and distal (> 100 μm) from amyloid plaques (Fig. 3a). All dynamic events between 3–6 MO and 6–9 MO were reported as a function of distance from the edge of the dense core amyloid plaques (Fig. 3b). ANOVA indicated spine eliminations concentrated closer to plaques (+ 46% 6–9 MO) whereas spine formations were higher in the intermediate zone (+ 194% 6–9 MO *P* < 0.02, Fig. 3c). Overall, spine eliminations outnumbered spine formations from 3 MO onwards in *Nr3c1*^ki/ki^ carriers (ratio 1.37 in *Nr3c1*^ki/ki^ *P* < 0.0001 and 3.3 in *Nr3c1*^ki/ki^-APP/PS1 *P* < 0.05, Additional file 1: Fig. S9a). This was reversed in *Nr3c1*^+/+^ (ratio 0.53 in controls *P* < 0.002) except in the cross with APP/PS1 mice (ratio 1.5, Additional file 1: Fig. S9b).

In terms of spine maintenance, ANOVA indicated significantly smaller rates close to amyloid plaques (*P* < 0.05, Fig. 3d). This applied to both the pre-existing spines (-11%) and newly formed spines (− 47%). Spine maintenance decreased at a faster rate in *Nr3c1*^ki/ki^ mice and APP/PS1 carrier compared to *Nr3c1*^+/+^ controls (− 5.2% of the pre-existing spines and -25.2% of the new spines, *P* < 0.05) (Additional file 1: Fig. S9b).

Spine clustering, which is defined as 2 or more formations (or eliminations) within 5 m of dendrite, is also critical for learning and memory [20]. We found clustered eliminations outnumbered clustered formations in APP/PS1 carriers at 9 MO (ratio 0.75 for *Nr3c1*^+/+^-APPS1 and 0.6 for *Nr3c1*^ki/ki^-APP/PS1) whereas it was equivalent in *Nr3c1*^+/+^ control mice (ratio 1.0, Additional file 1: Fig. S10). We compared the observed values with simulated clustering between a dynamic spine and any other on the dendrite to determine its probability. A t-test indicated spine clustering became random in *Nr3c1*^+/+^-APP/PS1 compared to *Nr3c1*^+/+^ littermates (*P* < 0.003). To determine if this observation is due to amyloid plaques, we compared the probability of spine clustering in the proximal and intermediate zones. As expected, clustered formations became random closer to amyloid plaques in both *Nr3c1*^+/+^-APP/PS1 and *Nr3c1*^ki/ki^-APP/PS1 genotypes (*P* < 0.05, Fig. 3e); clustered eliminations were random in the proximal and intermediate zones except for *Nr3c1*^ki/ki^-APP/PS1 mice (*P* = 0.04).

Collectively, we provide (i) a new topological map of spine dynamic attributes that is complementary to prior morphological studies in fixed tissue preparations [49], and (ii) evidence that loss of p-GR signaling contributes to AD-like neuroplasticity.

### Soluble Aβ42 oligomers affect p-GR status

**Fig. 4:**
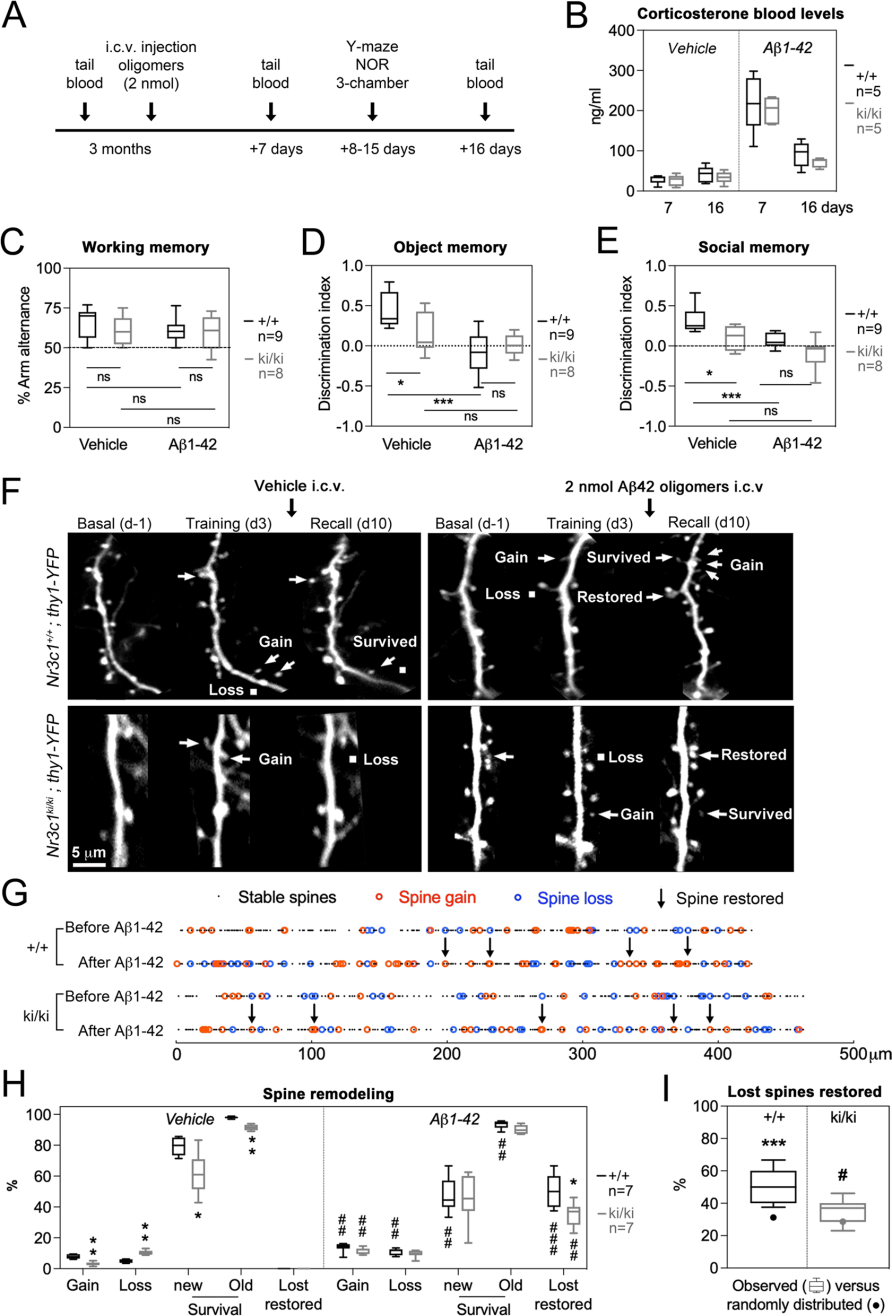
Soluble Aβ42 oligomers overlap with the effect of *Nr3c1*^ki/ki^ on spine maintenance. **a** Experimental timeline in NR3C1^ki/ki^-thy1-YFP double transgenic mice. **b** Corticosterone blood levels. Quartiles and median of dataset from Min-to-Max (N = 5 mice/group). Three-way ANOVA: effect of Aβ42 *F*_1,16_ = 123, *P* < 0.0001; effect of genotype *F*_1,16_ = 1.5, *P* = 0.2; effect of time *F*_1,16_ = 36.1, *P* < 0.0001 post-hoc Tukey test *P* < 0.05. **c** Percentage of alternance between arms of the Y-maze. Quartiles and median of dataset from Min-to-Max (N_(+/+,ki/ki)_ = 9,9 vehicle; 8,8 Aβ42 mice). Two-way ANOVA analyses show no effect of genotype or oligomers *P* > 0.05. **d** Time exploring the new object over the old one presented 24 h earlier in the novel object recognition test. Quartiles and median of dataset from Min-to-Max expressed as ratio index in N_(+/+,ki/ki)_ = 9,9 vehicle; 8,8 Aβ42 mice. Two-way ANOVA: effect of Aβ42 *F*_1,30_ = 20.1, *P* < 0.0001; interaction with *Nr3c1*^ki/ki^ *F*_1,30_ = 6.6, *P* = 0.01 post-hoc Tukey test *P < 0.05, ***P < 0.0001. **e** Time exploring the new mouse over the old one presented 24 h earlier in the 3-chamber test. Quartiles and median of dataset from Min-to-Max expressed as ratio index in N_(+/+,ki/ki)_ = 9,9 vehicle; 8,8 Aβ42 mice. Two-way ANOVA: effect of Aβ42 *F*_1,30_ = 17.8, *P* = 0.0002; effect of *Nr3c1*^ki/ki^ *F*_1,30_ = 13.5, *P* = 0.0009 post-hoc Tukey test *P < 0.05, ***P < 0.0001. **f** Dendritic spine remodeling in the motor cortex of mice trained on the rotarod then injected with soluble Aβ42 oligomers before recall of behavioral performance one week later. **g** Dynamic changes between images 1 and 2 (effect of training), and images 2 and 3 (effect of Aβ42). **h** Proportion of dendritic spines per categories of dynamic events between time points. Quartiles and median of dataset from Min-to-Max (N_(+/+,ki/ki)_ = 6,6 vehicle; 7,7 Aβ42 mice). Three-way ANOVA: effect of Aβ42 *F*_1,22_ = 15.78, *P* = 0.0006; effect of *Nr3c1*^ki/ki^ *F*_1,22_ = 14, effect of dynamic events *F*_4,88_ = 879, *P* < 0.0001, interaction of 3 factors *F*_4,88_ = 5.9, *P* = 0.0003. Multiple comparisons by Mann Whitney test between genotypes *P < 0.05, **P < 0.005 or between vehicle and Aβ42.^##^*P* < 0.001. **i** Restoration of lost spines is not random. Quartiles and median of dataset from Min-to-Max (N_(+/+,ki/ki)_ = 6,6 vehicle; 7,7 Aβ42 mice). Two-tailed unpaired t-test for comparing the observed versus 10,000 simulations of the restored lost spine at any position in the dendrite *P* < 0.05

The PS1^dE9^ mutation promotes the cleavage of APP^swe^ into Aβ42 that occurs in soluble forms before it deposits into plaques in APP/PS1 mice, modeling pathology in human familial AD brain [45]. To assess how soluble Aβ42 interacts with the *Nr3c1*^ki/ki^ genotype, we injected Aβ42 oligomers in cerebral ventricles of double transgenic *Nr3c1*^+/+^-thy1-YFP and *Nr3c1*^ki/ki^-thy1-YFP mice at 3 MO, and assessed neuroplasticity and memory retention a week later (Fig. 4a). Aβ42 increased corticosterone blood levels indistinguishably between genotypes. This effect dissipated with time (Fig. 4b) and was associated with impaired state of p-GR (Additional file 1: Fig. S11). To avoid the acute response to Aβ42 injection, memory performance was tested during the descending phase of corticosterone levels using the Y-maze (Fig. 3c), novel object recognition task and 3-chamber test. ANOVA indicated Aβ42 impaired non-social memory (− 120%, *P* < 0.0001, Fig. 4d) and social memory in *Nr3c1*^+/+^ mice (− 79%, *P* = 0.008, Fig. 4e) down to the levels of *Nr3c1*^ki/ki^ mice without additive effects (Fig. 4d, e).

To track the effects of soluble Aβ42 on task-activated neurons, we imaged dendritic spine dynamics in motor cortex evoked by rotarod training at 3 MO (Fig. 4f). Dynamic events between the 2nd and 3^rd^ views indicate how Aβ42 impacted the turnover and maintenance of spines cued to the task (Fig. 4g). ANOVA indicated Aβ42 oligomers increased spine formation in both genotypes (+ 74% for *Nr3c1*^+/+^ and + 360% for *Nr3c1*^ki/ki^, *P* < 0.01), and increased spine elimination in *Nr3c1*^+/+^ mice (+ 110%, *P* = 0.008) to the levels of *Nr3c1*^ki/ki^ mice, similar to observations in APP/PS1 carriers. Aβ42 also decreased the survival of training-induced spines down to the level of *Nr3c1*^ki/ki^ mice (-42% compared to -38% for *Nr3c1*^+/+^, *P* < 0.0001, Fig. 4h), again similar to observations in APP/PS1 mice at 6–9 MO. A surprising number of spines lost upon training reappeared after injection of Aβ42 in both genotypes (51% *Nr3c1*^+/+^ and 34% *Nr3c1*^ki/ki^, *P* < 0.0001, Fig. 4h). A t-test indicated restoration of lost spines was significantly different from de novo spine formation in *Nr3c1*^+/+^ mice (*P* = 0.012) and random in *Nr3c1*^ki/ki^ mice (*P* = 0.7, Fig. 4i). Together, these results indicate that loss of p-GR signaling overlapped with soluble Aβ42 to eliminate spine dynamic subtypes known to retain memory.

### Altered p-GR signaling impairs synaptic plasticity and AMPA receptors mobilization in task-induced spines

**Fig. 5:**
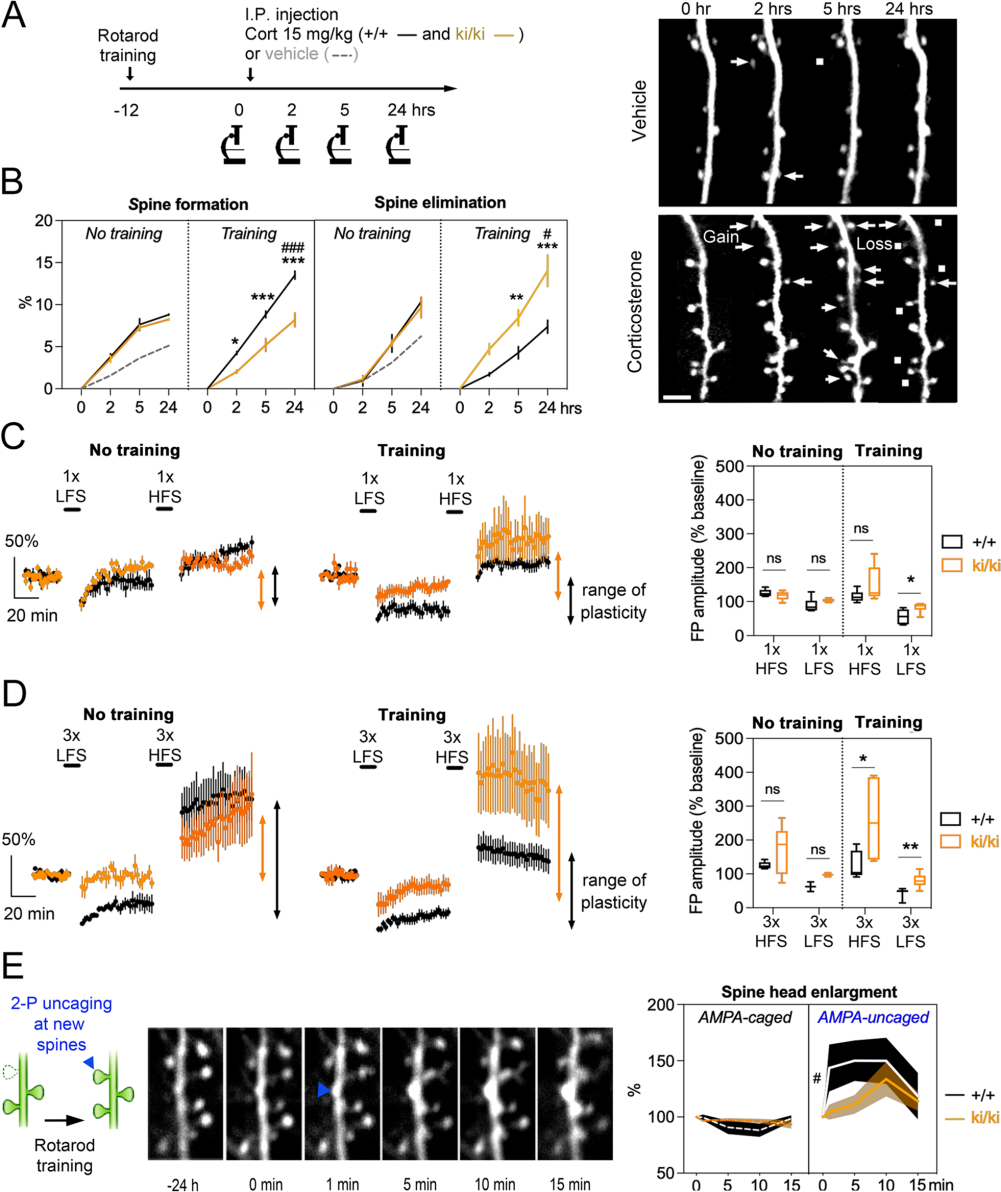
Lack of p-GR decreases synaptic plasticity and the mobilization of AMPA receptors in task-induced dendritic spines. **a** Experimental timeline in *Nr3c1*^+/+^-thy1-YFP and *Nr3c1*^ki/ki^-thy1-YFP double transgenic mice and timelapse imaging in motor cortex. **b** Interaction of training and genotype on spine formation and elimination. Means ± SEM of N_(+/+, ki/ki)_ = 4,4 mice without and 6,5 with training. Three-way ANOVA: effect of genotype on formation *F*_1,59_ = 55, *P* < 0.0001; effect of time post-CORT injection on formation *F*_3,59_ = 422, *P* < 0.0001; effect of training on formation *F*_1,59_ = 2.7, *P* = 0.1; effect of genotype on elimination *F*_1,61_ = 17.9, *P* < 0.0001; effect of time post-CORT injection on elimination *F*_3,61_ = 129, *P* < 0.0001; effect of training on elimination *F*_1,61_ = 5.8, *P* = 0.01 post-hoc Tukey test comparing genotypes *P = 0.01, **P = 0.007, ***P < 0.0001 or training groups ^#^*P* < 0.02, ^###^*P* < 0.0001. **c** Field potentials (Means ± SEM) recorded in acute slices of motor cortex on the next day of rotarod training. High-frequency stimulation (HFS) and low–frequency stimulation (LFS) with half the intensity to reach a maximal response in parallel fibers placed ≈500 μm away from the recording electrodes induced LTP and LTD, respectively. Quartiles and median of dataset from Min-to-Max (N_(+/+,ki/ki)_ = 5,6 HFS; 8,5 LFS mice without training and 5,6 HFS; 9,6 LFS with training). Mann–Whitney test comparing genotypes *P < 0.01. **d** Protocol of metaplasticity with 3 consecutive HFS or LFS interspaced with at least 20-min intervals to return to baseline activity. Quartiles and median of dataset from Min-to-Max (N_(+/+,ki/ki)_ = 5,5 HFS; 3,3 LFS mice without training and 8,6 HFS; 8,6 LFS with training). Mann–Whitney test comparing genotypes *P = 0.029, **P = 0.008. **e** Two-photon uncaging of AMPA at newly formed dendritic spines on the next day of rotarod training. Spine head enlargement normalized to the size prior AMPA uncaging (blue arrow). Means ± SEM of N_(+/+, ki/ki)_ = 13,15 spines without and 18,33 with uncaging. Three-way ANOVA: effect of genotype *F*_1,340_ = 4 *P* = 0.04; effect of uncaging *F*_1,340_ = 23.7 *P* < 0.0001 post-hoc Tukey comparing genotype and uncaging.^#^*P* = 0.02

GR phosphorylation signaling output depends on 2 pathways, the BDNF and cortisol. Both pathways must be activated to unravel its mechanistic impact. We used a 2-hit model in which cortisol is injected systemically and BDNF is secreted locally by the activity-dependent pathway triggered by rotarod training as described [2]. For this experiment, we used double transgenic mice *Nr3c1*^+/+^-thy1-YFP and *Nr3c1*^ki/ki^-thy1-YFP to isolate the role of p-GR deficit from the overlapping amyloid-β pathway. We trained mice at 3 MO and determined the temporal dynamics of dendritic spine turnover (Fig. 5a). We find genotype interacted with training to promote corticosterone-evoked spine formation (*P* = 0.001) and elimination (*P* = 0.01) in differential time domains. In the absence of training, the ratio of spine formation:elimination was 0.86 for *Nr3c1*^+/+^ and 0.85 for *Nr3c1*^ki/ki^ (Fig. 5b). In contrast, training altered the rates between genotypes (*P* < 0.01) by increasing spine formation (*P* = 0.001) and decreasing spine elimination in *Nr3c1*^+/+^ mice (*P* = 0.01). This was opposite in *Nr3c1*^ki/ki^ mice (*P* = 0.02). Thus, GR mutations impeded the remodeling of dendritic spines that is expected from behavioral training and cortisol rising levels.

Functionally, we evoked long-term potentiation (LTP) and long-term depression (LTD) in ex-vivo slices of motor cortex on the next day of rotarod training. GR mutations impaired the expression of LTD only in trained cortices (*P* < 0.01) without altering LTP (Fig. 5c). We also triggered metaplasticity by applying consecutive protocols (3 ×) of low or high frequency stimulation because it reveals the endogenous state of plasticity triggered by training within the range of ex-vivo plasticity as previously described [46]. In this context, GR mutations impaired the expression of both LTP (*P* = 0.029) and LTD (*P* < 0.001) in trained cortices (Fig. 3d). Further application the GABA receptor antagonist bicuculline revealed ectopic activity in mice lacking p-GR sites compared to littermate controls (Additional file 1: Fig. S12). We conclude the in-vivo synaptic plasticity triggered by training is restrained in mice lacking p-GR sites.

To bridge the gap between p-GR, training, spine plasticity and dynamics, we performed 2-photon uncaging of the previously described caged-AMPA [42] specifically at task-induced spines at 3 MO because GluA1 surface expression and synaptosomal content are reduced in mice lacking p-GR sites [2]. To this end, we used transcranial microscopy before and after the training to identify the newly formed spines; and then opened the skull and meninges to deliver caged-AMPA in the region of interest. Laser stimulation directly at the spine head provoked a typical enlargement within minutes (Fig. 5e). ANOVA indicated the specific effect of AMPA uncaging (*P* < 0.0001) as well as an effect of genotype on the response kinetics (max at ~ 5 min for *Nr3c1*^+/+^ and ~ 10 min for *Nr3c1*^ki/ki^, *P* = 0.02) more than amplitude (+ 50% max for *Nr3c1*^+/+^ and + 33% max for *Nr3c1*^ki/ki^, *P* > 0.05). Taken together, these data indicate BDNF-dependent p-GR signaling is necessary for task-induced synaptic plasticity and suppressed by amyloid-β.

## Discussion

Amyloid-β as insoluble and soluble forms harms groups of dendritic spines known to retain memory. We provide evidence that BDNF-mediated p-GR signaling pathway counteracts these effects and strengthens neuroplasticity and memory retention since its genetic disruption accelerates deficits in memory and synaptic plasticity similar to AD-prone models. This is consistent with low abundance of BDNF-dependent p-GR and high-level cortisol-dependent p-GR in cortex of AD subjects. Therefore, GR signaling is beneficial when BDNF and cortisol levels are paired, and detrimental in AD when unpaired [22, 41]. The phosphorylation of GR intrinsically disordered domain is expected to stabilize docking sites for signaling effectors dissociating the beneficial from deleterious effects of cortisol in AD.

We found that expression of p-GR isoforms induced by the BDNF pathway is brain and neuronal specific unlike cortisol-dependent p-GR pathway that is more widely distributed among tissues and cell types. For instance, glia express dominant negative truncated TrkB such that p-GR at cortisol-dependent sites are exclusive in these cells. This makes BDNF-dependent p-GR signaling an attractive target for AD dementia.

We also report for the first time a functional interaction between the APP/PS1 model of AD and impaired BDNF-dependent p-GR signaling triggered a neurocentric phenotype, without altering vascular pathology and amyloid-β deposition [16]. Despite the expected primary neurocentric effects of GR mutations, secondary indirect effects could make neurons more susceptible to the damaging effects of amyloid-β pathways (e.g. inflammation). We find impaired neuroplasticity evoked by p-GR status involve synaptic surface expression of AMPA receptors. This is consistent with our previous study linking BDNF-dependent p-GR to increased trafficking of AMPA receptors in synaptosomes [2] and related studies pointing at the role of new inserted AMPA receptors on synaptic plasticity and memory [32].

We also present evidence that soluble Aβ42 injected into the mouse brain reduced p-GR at the BDNF- but not cortisol-dependent sites. This is consistent with the interaction between NR3C1^ki/ki^ genotype and both soluble Aβ42 and insoluble plaques to disrupt dendritic spine plasticity. Indeed, both the *Nr3c1*^ki/ki^ and APP/PS1 mice showed striking similarities in net spine loss, reduced spine survival and decreased spine clustering. One key difference is the topology of these events ruled by the depositions of amyloid-β surrounded by a halo of soluble Aβ42 [49]. The spine attrition focus at plaques surrounded by a halo of spinogenesis is consistent with the abnormally high proportions of silent neurons proximal to plaques and hyperactive neurons in its surroundings [13]. Therefore, the distribution of amyloid-β as plaques and oligomers could alter neuronal connectivity in ways that could transform how the memory trace form and evolve with time.

Identifying subtypes of dendritic spines affected by amyloid-β is important to understand its impact on memory. We defined subtypes based on dynamics rather than morphology because spine turnover occurs specifically in task-activated dendrites [15]. Neighboring dendritic spines that cluster are typically responsive to distinct stimuli [31], and may retain the functional connectivity from inputs signaling the associative contents of memory. Maintenance of these clusters depends on local mitochondrial metabolism influenced by stress and BDNF levels [18], in animal models of AD [52] and in human AD brain [51]. The restoration of task-related lost spines promoted by the combination of soluble Aβ42 and reduced BDNF-dependent p-GR signaling has never been seen before in AD models. This is consistent with the restoration of lost spines that occurs in depression models after successful antidepressant therapy [40], and relies on the normalization of BDNF and cortisol levels [12, 27]. These findings argue for the beneficial effects of BDNF-dependent GR pathway over the detrimental effects of the cortisol-dependent GR pathway.

As amyloid-β accumulates in the brain, aberrant turnover and connectivity of task-related dendritic spine clusters could underlie the loss of memory accuracy [1]. Since targeting amyloid-β has yet to be successful in treating AD [29], alternative approaches that effectively improve damaged neuronal connectivity, such as *promoting* the BDNF-dependent GR pathway, could help reverse cognitive decline in people with AD and provide a novel avenue of effective therapeutics.

## Supplementary Information

**Additional file 1**. Supplementary figures and legends.

## Data Availability

The French ministry of research and ethics committee CEEA36 approved the protocols adhering to the 2010/63/UE directive of the European community for the care and use of laboratory animals.
