# Supplementary material for: Loss of glucocorticoid receptor phosphorylation contributes to cognitive and neurocentric damages of the amyloid-β pathway
Source: Acta Neuropathol Commun. 2022 Jun 22;10:91. doi: 10.1186/s40478-022-01396-7 (PMC9219215; doi:10.1186/s40478-022-01396-7)
Supplement: Supplementary file 1 — Additional file 1. Supplementary figures and legends. [file 40478_2022_1396_MOESM1_ESM.pdf]

Additional File 1: Supplementary Figures

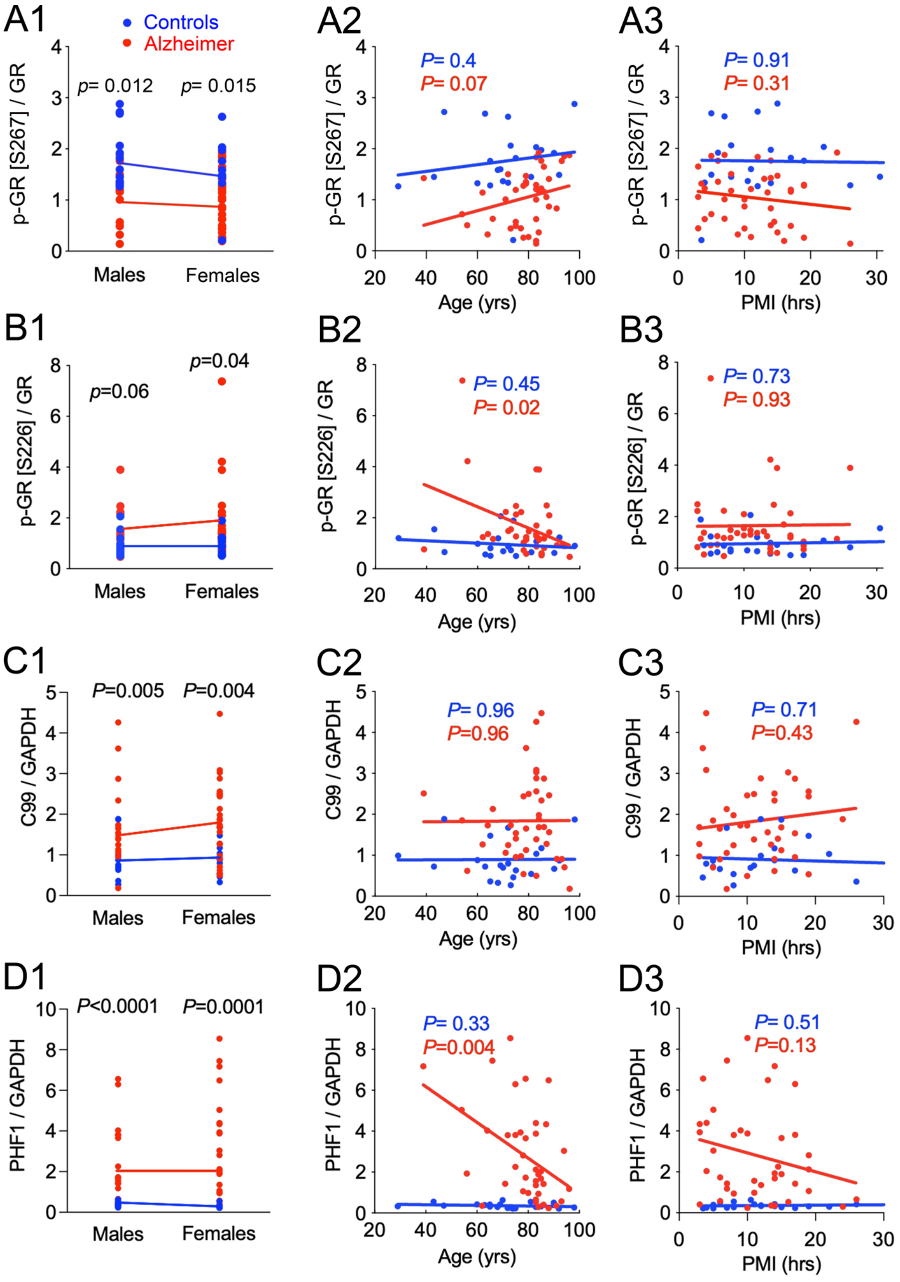

Fig. S1. Interaction of protein expression with demographic factors

A1. No effect of gender on the expression of p-GR[S267]. Lines show the mean. Kruskal-Wallis test in men (N=12 C, 14 A *P*=0.012) and women (N=9 C, 26 A *P*=0.015).

A2. Effect of age on the expression of p-GR[S267]. Coefficients of Pearson in 21 controls (r =0.17) and 40 AD cases (r =0.29) indicated that parameters are not correlated.

A3. Effect of postmortem interval on the expression of p-GR[S267]. Coefficient of Pearson in 21 controls (r=-0.02) and 40 cases (r=-0.16) indicated that parameters are not correlated.

B1. Effect of gender on the expression of p-GR[S226]. Lines show the mean. Kruskal-Wallis test in men (N=12 C, 14 A, *P*=0.06) and women (N=9 C, 26 A, *P*=0.04). Data were more powered in females than males likely due to the sample size.

B2. Effect of age on the expression of p-GR[S226]. Coefficients of Pearson in 21 controls (r=-0.17, *P*=0.45) and 40 AD cases (r=-0.37, *P*=0.02). Data were more powered in the aged subjects than the young. Even though p-GR[S226] levels decreased with age in the AD group, it cannot explain the overall increase of p-GR[S226] in cases compared to controls.

B3. No effects of PMI on the expression of p-GR[S226]. Coefficient of Pearson in 21 controls (r =0.07) and 40 AD cases (r =0.013).

C1. No effect of gender on the expression of C99 fragment. Lines show the mean. Kruskal-Wallis test in men (N=12 C, 14 A, *P*=0.005) and women (N=9 C, 26 A, *P*=0.004).

C2. No effect of age on the expression of C99 fragment. Coefficients of Pearson in 21 controls (r=0.009, *P*=0.96) and 40 AD cases (r=0.006, *P*=0.96).

C3. No effects of PMI on the expression of C99 fragment. Coefficient of Pearson in 21 controls (r =-0.08, *P*=0.71) and 40 AD cases (r =0.12, *P*=0.43).

D1. No effect of gender on the expression of PHF-1. Lines show the mean. Kruskal-Wallis test in men (N=12 C, 14 A, *P*<0.0001) and women (N=9 C, 26 A, *P*=0.0001).

D2. Effect of age on the expression of PHF-1. Coefficients of Pearson in 21 controls (r=-0.22, *P*=0.33) and 40 AD cases (r=-0.43, *P*=0.0046).

D3. No effects of PMI on the expression of PHF-1. Coefficient of Pearson in 21 controls (r =0.15, *P*=0.51) and 40 AD cases (r =-0.23, *P*=0.13).

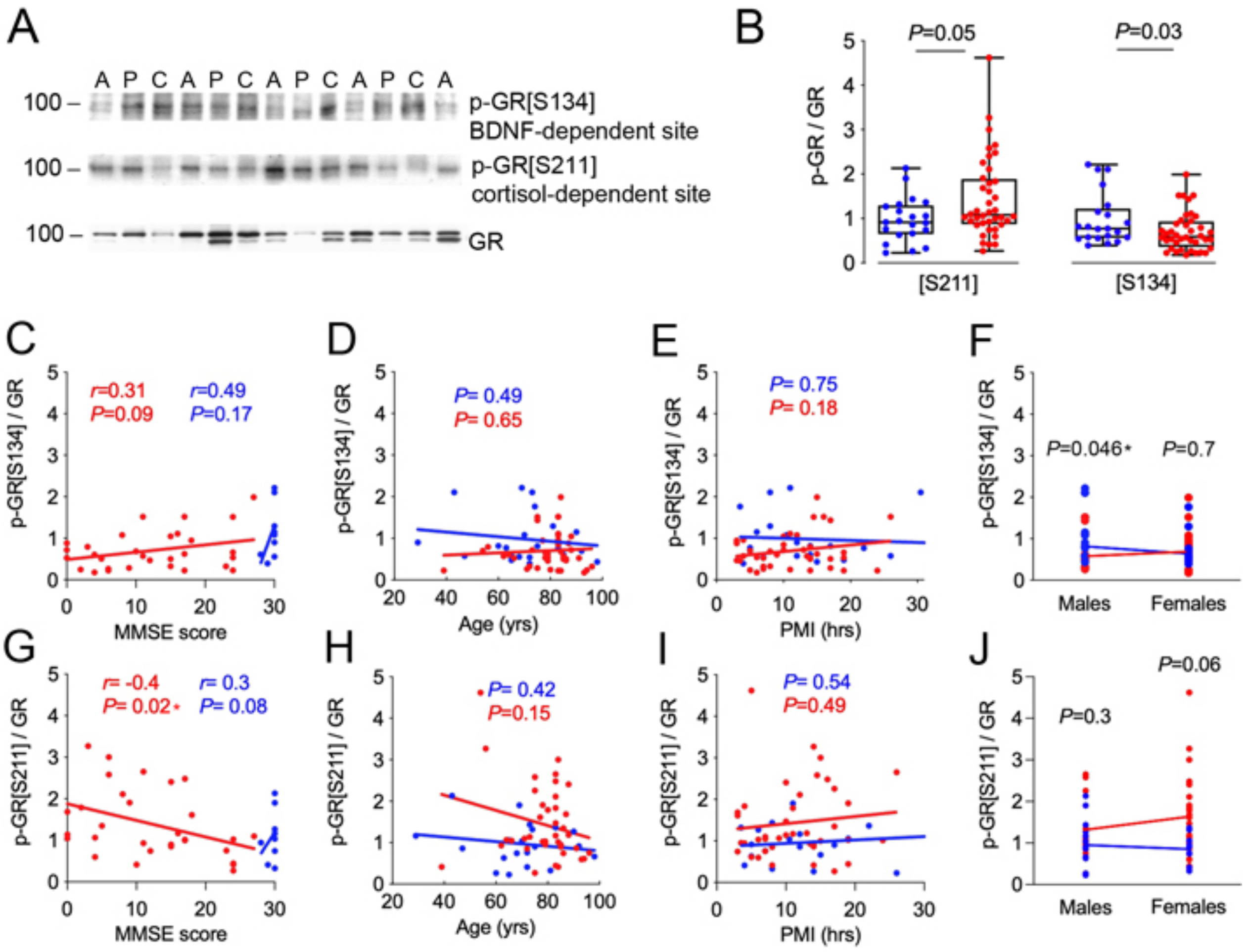

Fig. S2. Validation of p-GR status at other sites in AD brains

A. Immunodetection of proteins in the prefrontal cortex (C, controls, A, Alzheimer, P, Parkinson).

B. Ratio of optical densities for GR and p-GR at the other BDNF-responding site S134 and another glucocorticoid-responding site S211. Quartiles and median of dataset from Min-to-Max. Significant differences with Mann-Whitney test between 21 C and 40 A is absent between 21 C and 18 P (*P*=0.03 for S134 and *P*=0.05 for S211).

C. Effect of MMSE score on the expression of p-GR[S134]. Coefficients of Pearson in 21 controls (r=0.31, *P*=0.09) and 40 AD cases (r=0.49, *P*=0.17). There is only a trend for a decreased expression in AD.

D. No effect of age on the expression of p-GR[S134]. Coefficients of Pearson in 21 controls (r=0.07, *P*=0.65) and 40 AD cases (r=-0.15, *P*=0.49).

E. No effects of PMI on the expression of p-GR[S134]. Coefficient of Pearson in 21 controls (r =-0.07, *P*=0.75) and 40 AD cases (r =0.21, *P*=0.18).

F. Effect of gender on the expression of p-GR[S134]. Lines show the mean. Kruskal-Wallis test in men (N=12 C, 14 A, *P*=0.046) and women (N=9 C, 26 A, *P*=0.7). Data were more powered in females than males likely due to the sample size.

G. Effect of MMSE score on the expression of p-GR[S211]. Coefficients of Pearson in 21 controls (r=0.29, *P*=0.4) and 40 AD cases (r=-0.4, *P*=0.028). There is an increase of expression in AD.

H. No effect of age on the expression of p-GR[S211]. Coefficients of Pearson in 21 controls (r=-0.18, *P*=0.42) and 40 AD cases (r=-0.22, *P*=0.15).

I. No effects of PMI on the expression of p-GR[S211]. Coefficient of Pearson in 21 controls (r =-0.14, *P*=0.54) and 40 AD cases (r =0.11, *P*=0.49).

J. No effect of gender on the expression of p-GR[S211]. Lines show the mean. Kruskal-Wallis test in men (N=12 C, 14 A, *P*=0.3) and women (N=9 C, 26 A, *P*=0.06).

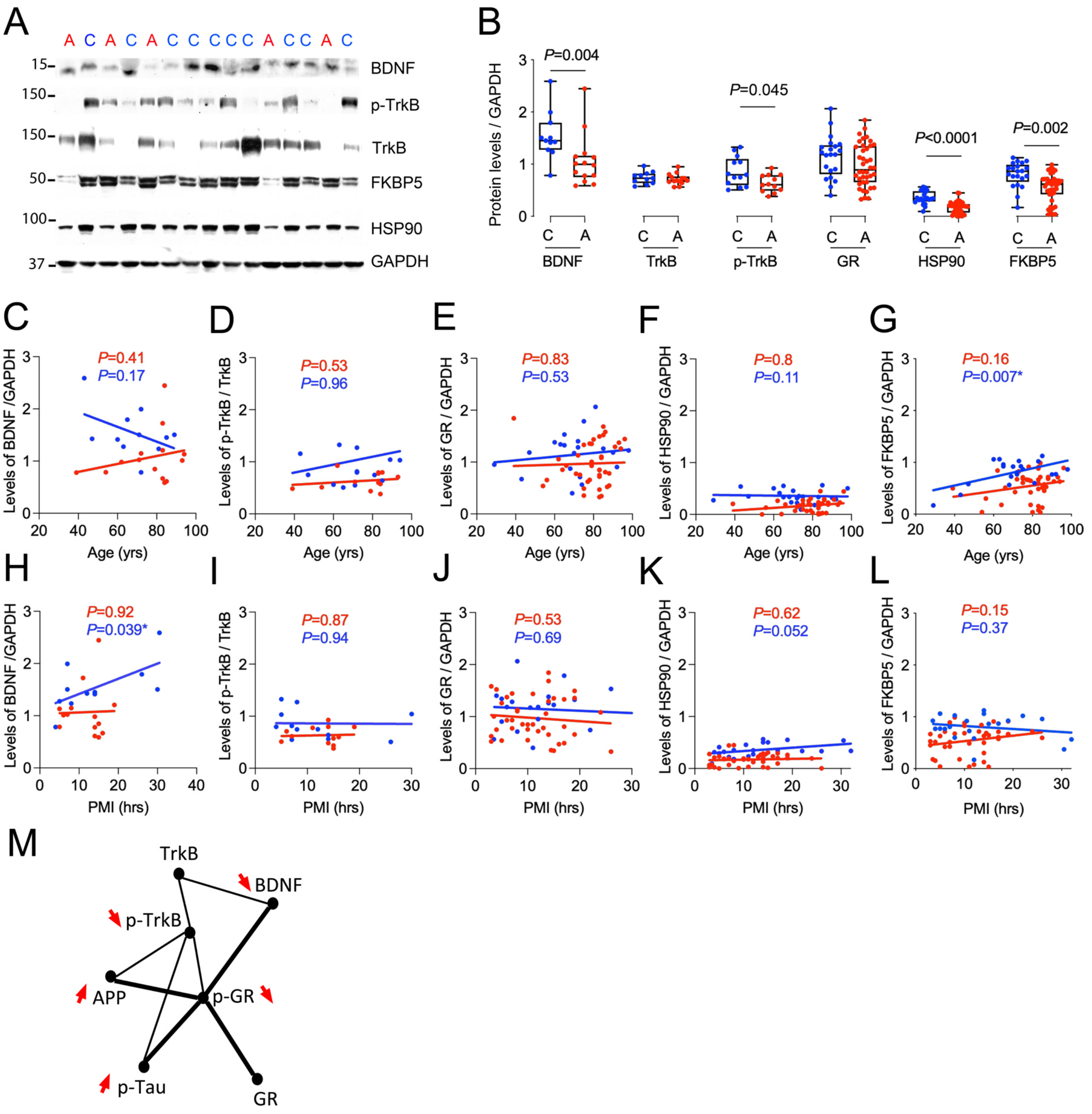

Fig. S3. Expression of BDNF signaling modules in AD brains

A. Expression of proteins interacting functionally with GR.

B. Ratio of optical densities for candidate proteins over GAPDH. Quartiles and median of dataset from Min-to-Max. Significant difference with Mann-Whitney test as indicated.

C. No effect of age on the expression of BDNF. Coefficients of Pearson in 11 controls (r=-0.18, *P*=0.17) and 14 AD cases (r=-0.22, *P*=0.41).

D. No effect of age on the expression of p-TrkB/TrkB. Coefficients of Pearson in 13 controls (r=-0.01, *P*=0.9) and 12 AD cases (r=-0.19, *P*=0.53).

E. No effect of age on the expression of GR. Coefficients of Pearson in 21 controls (r=0.14, *P*=0.53) and 40 AD cases (r=0.03, *P*=0.8).

F. No effect of age on the expression of HSP90. Coefficients of Pearson in 21 controls (r=-0.05, *P*=0.8) and 40 AD cases (r=0.13, *P*=0.11).

G. Effect of age on the expression of FKBP51. Coefficients of Pearson in 21 controls (r=0.56, *P*=0.007) and 40 AD cases (r=0.22, *P*=0.15). There is an effect of age on the levels of FKBP51.

H. Effects of PMI on the expression of BDNF. Coefficient of Pearson in 11 controls (r =0.02, *P*=0.9) and 14 AD cases (r =0.62, *P*=0.04). There is an effect of PMI in AD samples.

I. No effects of PMI on the expression of p-TrkB/TrkB. Coefficient of Pearson in 13 controls (r =-0.019, *P*=0.9) and 12 AD cases (r =0.05, *P*=0.8).

J. No effects of PMI on the expression of GR. Coefficient of Pearson in 21 controls (r =-0.09, *P*=0.69) and 40 AD cases (r =-0.07, *P*=0.53).

K. No effects of PMI on the expression of HSP90. Coefficient of Pearson in 21 controls (r =0.42, *P*=0.052) and 40 AD cases (r =0.07, *P*=0.62).

L. No effects of PMI on the expression of FKBP51. Coefficient of Pearson in 21 controls (r =-0.2, *P*=0.15) and 40 AD cases (r =0.23, *P*=0.15).

M. Reduction of the BDNF-GR signaling pathway in AD neuropathology.

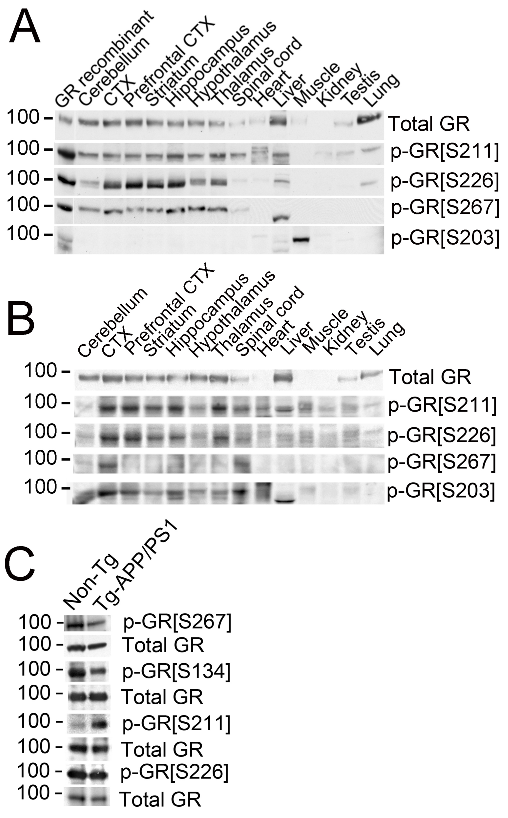

Fig. S4. Brain-specific expression of GR phospho-isoform responding to BDNF and in APP/PS1 mice

A. Several tissues from a non-transgenic mouse (8 months-old) were chopped and suspended in RIPA buffer. Lysates were cleared from debris by centrifugation and proteins dosed using the BCA methods against known concentrations of BSA. Equal amounts of proteins (50 μg) were loaded in each lane of a 10% SDS-PAGE and blotted onto PVDF membranes for detection with antibodies against p-GR at the BDNF-responding site and the cortisol-responding ones (S203, S211, S226). Apart from one band migrating at the wrong size in the liver, the p-GR[S267] antibody detected its target only in tissues of the central nervous system. The p-GR[S226] and p-GR[S211] antibodies detected its target in both CNS and peripheral tissues. On the contrary, the p-GR[S203] antibody detected its target only in peripheral tissues.

B. Tissue expression of p-GR isoforms in 8 months old APP/PS1 mouse (50 μg protein loaded).

C. Lysates (50 μg) of prefrontal cortex dissected from transgenic APP/PS1 (Tg) mice and non-transgenic controls at 8 months of age were separated in a 10% SDS-PAGE and immunoblotted with the indicated antibodies.

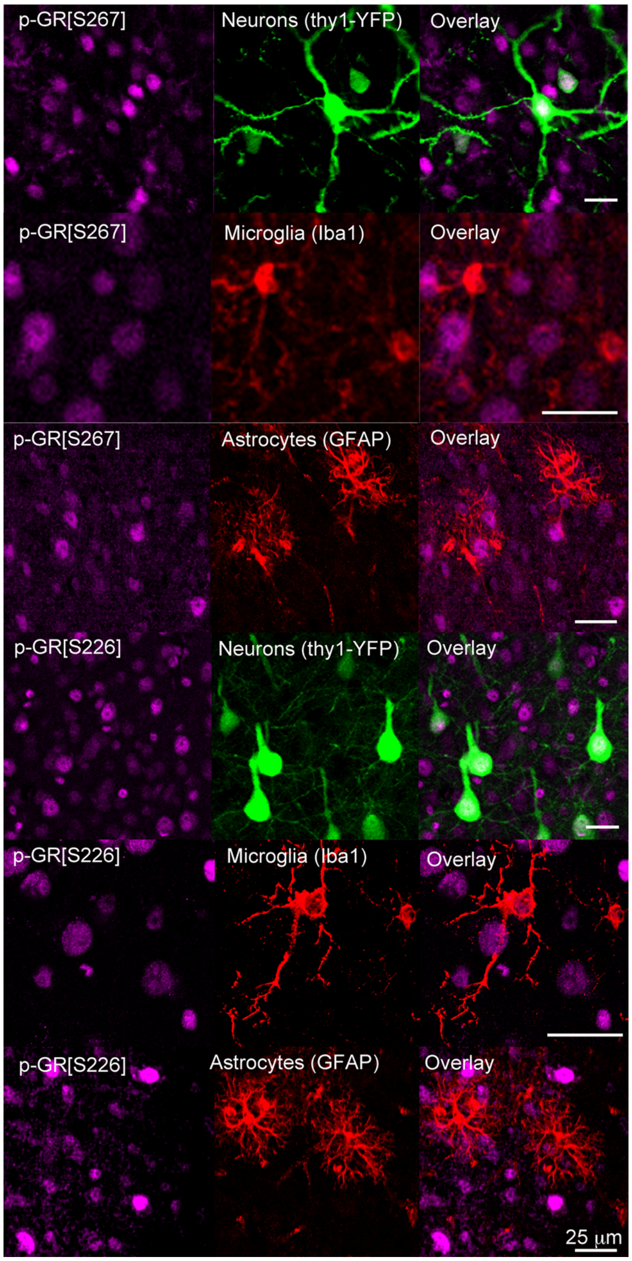

Fig. S5. BDNF-dependent p-GR isoform is most abundant in neurons

Coronal sections of mouse brain stained with antibodies against the cortisol-dependent p-GR[S226] or the BDNF-dependent p-GR[S267] together with markers of microglia (Iba1), astrocytes (GFAP) and excitatory neurons (thy1-YFP). Scale =25 μm.

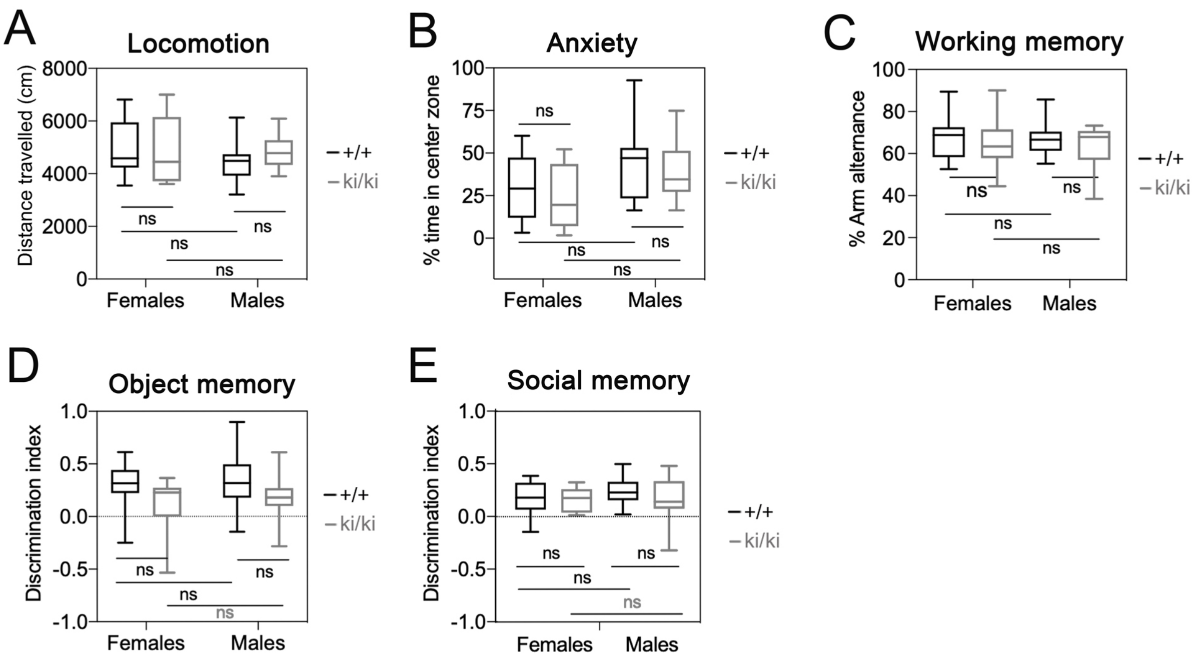

Fig. S6. Behavioral sex differences in knockin mice lacking GR phosphorylation at BDNF-responding sites at 3 months of age

A. Total distance travelled in the open field. Quartiles and median of dataset from Min-to-Max (N=12 males and 10 females/group). Two-way ANOVA analyses show no effect of gender $F_{1,40}$=0.8 and genotype $F_{1,40}$=0.3, P>0.1.

B. Time spent in the center zone of the open field. Quartiles and median of dataset from Min-to-Max (N=12 males and 10 females/group). Two-way ANOVA: effect of gender $F_{1,40}$=7.2 P=0.01, genotype $F_{1,44}$=1 P>0.05 post-hoc Tukey test P>0.05.

C. Percentage of alternance between arms of a Y-maze. Quartiles and median of dataset from Min-to-Max (N=12 males and 10 females/group). Two-way ANOVA: effect of gender $F_{1,40}$=0.2, genotype $F_{1,44}$=1.2 P>0.05.

D. Time exploring the new object over the old one presented 24 hrs earlier in the novel object recognition test. Quartiles and median of dataset from Min-to-Max expressed as ratio index in N=12 males and 10 females/group. Two-way ANOVA: effect of gender $F_{1,40}$=0.2; genotype $F_{1,40}$=5, P=0.02 post-hoc Tukey test P>0.05.

E. Time exploring the new mouse over the old one presented 24 hrs earlier in the 3-chamber test. Quartiles and median of dataset from Min-to-Max expressed as ratio index in N=12 males and 10 females/group. Two-way ANOVA: effect of gender $F_{1,40}$=0.3; genotype $F_{1,40}$=0.9 P>0.05.

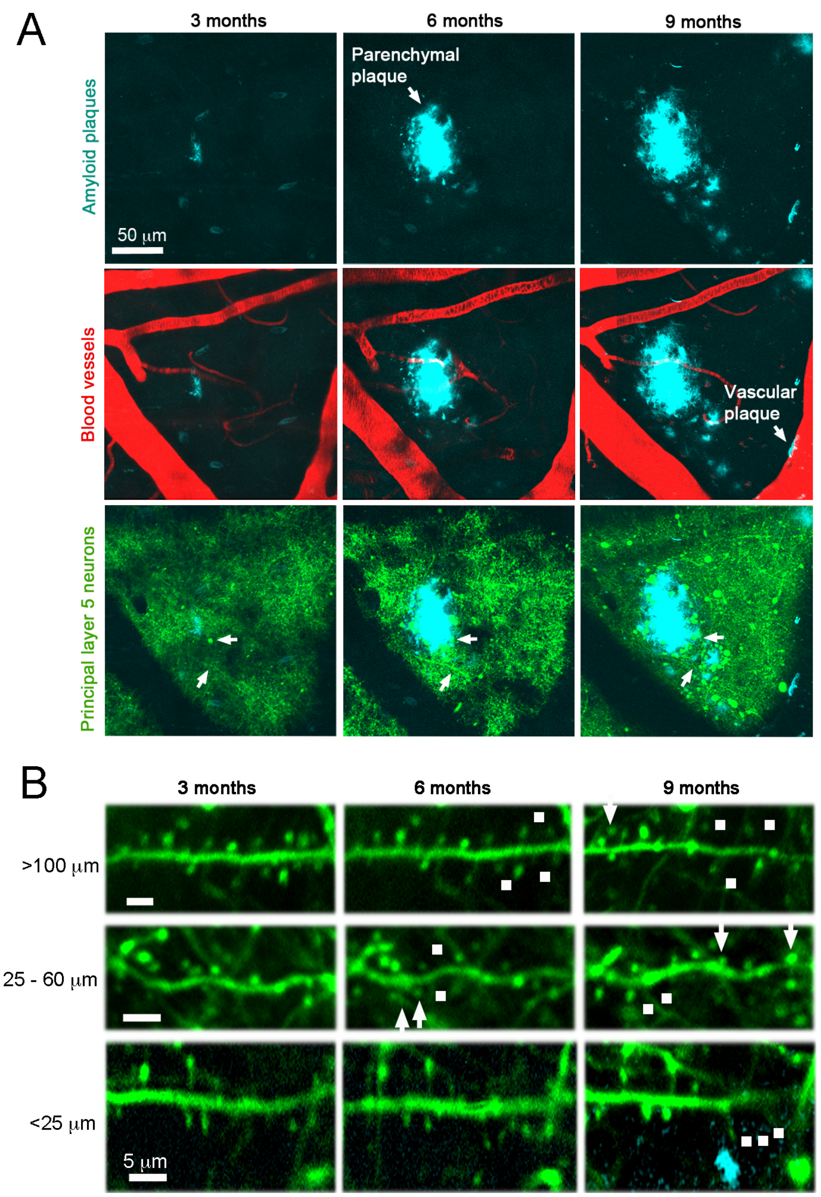

SI-Fig. S7. Transcranial imaging of *Nr3c1*$^{ki/ki}$ mouse

A. Repeat images of a cortical volume in somatosensory cortex at 3, 6 and 9 MO to track in 3D the amyloid plaques (methoxy-XO4), blood vessels (75 KDa dextran-AlexA594) and pyramidal neurons of layer 5 (thy1-YFP).
B. Dendritic spine remodeling in 3 zones based on distance from the nearest amyloid plaque: the proximal (< 25 μm), intermediate (25-60 μm) and distal (>100 μm). Squares indicate spine losses while arrows point to spine gains.

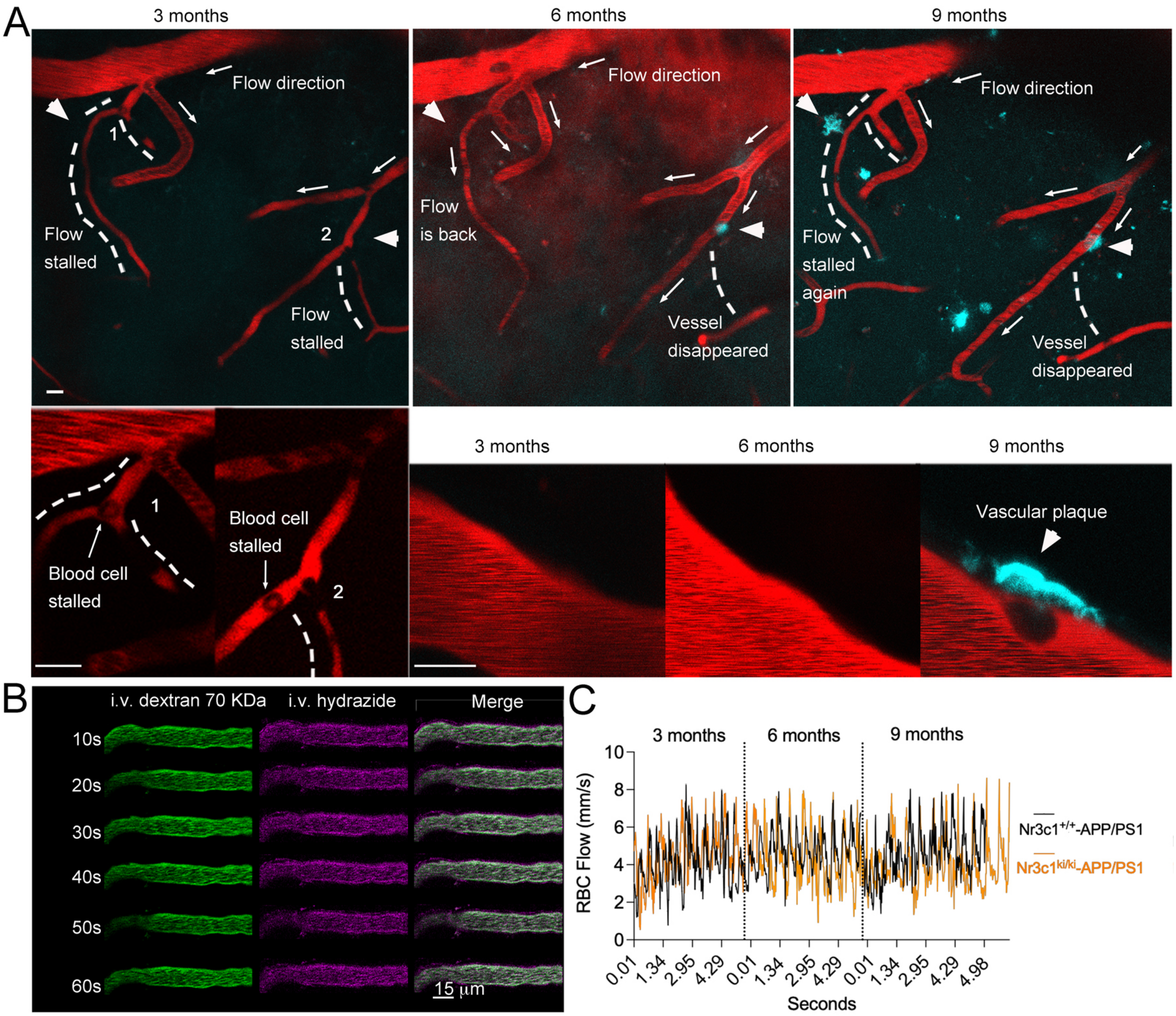

Fig. S8. Imaging the flow and adhesion of blood cells

A. Blood flow interrupted by cells stalling at vessels bifurcation (see zoom 1 and 2 for flow stalling where β-amyloid plaque will appear). This phenomenon can be reversible or associated with the disappearance of the vessel between sessions. Inset shows a blood cell adhering to the vessel wall at the site of vascular β-amyloid deposits. Scale =10 μm.

B. Arterioles specially labeled with hydrazide-Alexa633 injected i.v. 24 hrs prior imaging and RBC velocity using Alexa-FITC-dextran 70Kda injected i.v. just before imaging.

C. RBC flow was analyzed using an in house Matlab sofware (see methods). Matlab code for Line-Scanning Particle Image Velocimetry available at: https://sourceforge.net/projects/lspivsupplement/files. One line was traced along the center lumen of the vessel. Parallel line-scans were automatically traced from the reference line and the

emission fluorescence along this ribbon along the vessel was averaged to give RBC flow in pixels/frames converted as mm/s. We selected arterioles of a diameter of ~10 μm since flow depends on caliber. Flow rate is the average in a period of 5 sec to encompass several heart beat pulsations. Flow is not different between time points and genotypes.

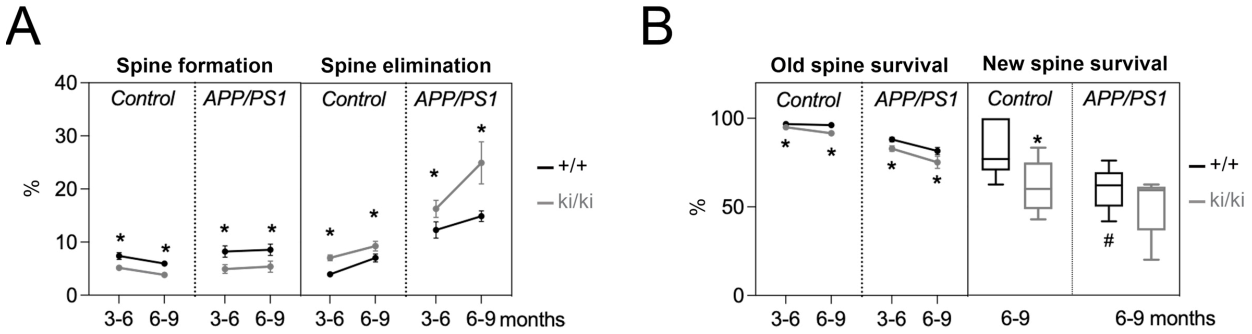

Fig. S9. Effect of APP/PS1 overlaps with that of NR3C1$^{ki/ki}$ genotype on spine maintenance

A. Quantitative dynamics of dendritic spines in somatosensory cortex. Means±SEM of $N_{(3,6,9\ months)}$=8,8,8 NR3C1$^{+/+}$, 9,9,9 NR3C1$^{ki/ki}$, 9,9,7 NR3C1$^{+/+}$-APP/PS1, 9,9,5 NR3C1$^{ki/ki}$-APP/PS1 mice. Three-way ANOVA: effect of APP/PS1 on formation $F_{1,56}$=5.2 and elimination $F_{1,60}$=120, P<0.02; effect of NR3C1$^{ki/ki}$ on formation $F_{1,56}$=26.9 and elimination $F_{1,60}$=26.6, P<0.0001; effect of aging on formation $F_{1,56}$=0.9 and elimination $F_{1,60}$=26.2, P<0.0001 post-hoc Tukey test *p<0.05.

B. Survival of dendritic spines in somatosensory cortex. Old spines (means±SEM) of $N_{(3,6,9\ months)}$=8,8,8 NR3C1$^{+/+}$, 9,9,9 NR3C1$^{ki/ki}$, 9,9,7 NR3C1$^{+/+}$-APP/PS1, 9,9,5 NR3C1$^{ki/ki}$-APP/PS1 mice. Three-way ANOVA: effect of APP/PS1 $F_{1,56}$=145, P<0.0001; effect of NR3C1$^{ki/ki}$ $F_{1,56}$=19.4, P<0.0001; effect of aging $F_{1,56}$=17.7, P<0.0001 post-hoc Tukey test *p<0.05. New spines (quartiles and median of dataset from Min-to-Max) of $N_{(+/+,\ ki/ki,\ +/+;APP/PS1,\ ki/ki;APP/PS1)}$=7, 8, 5, 5 mice. Two-way ANOVA: effect of APP/PS1 $F_{1,22}$=7.1, P=0.013; effect of NR3C1$^{ki/ki}$ $F_{1,22}$=6.5, P=0.018 post-hoc Tukey test comparing NR3C1$^{+/+}$ with NR3C1$^{ki/ki}$ *p<0.005, and NR3C1$^{+/+}$ with NR3C1$^{+/+}$-APP/PS1 or with NR3C1$^{ki/ki}$-APP/PS1 $^{\#}p$<0.05.

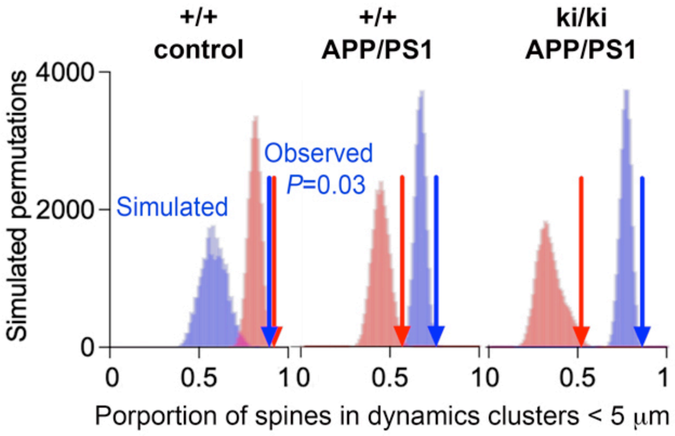

Fig. S10. Spine clustering is different from chance levels in NR3C1$^{+/+}$ controls unlike in the APP/PS1 mice

Observed (Mean of dataset pointed by arrows) versus simulated (Gaussian fit) clustering of dynamic spines (red=gains and blue=losses). $N_{(+/+,\ +/+;APP/PS1,\ ki/ki;APP/PS1)}$=6, 8, 8 mice. Two-way ANOVA: effect of genotype on gains $F_{2,36}$=28.3, P<0.0001 and losses $F_{2,37}$=4, P=0.02; effect of clustering on gains $F_{1,36}$=6.1, P=0.01 and losses $F_{1,37}$=15, P=0.0004 post-hoc Tukey test comparing observed vs simulated *p=0.03.

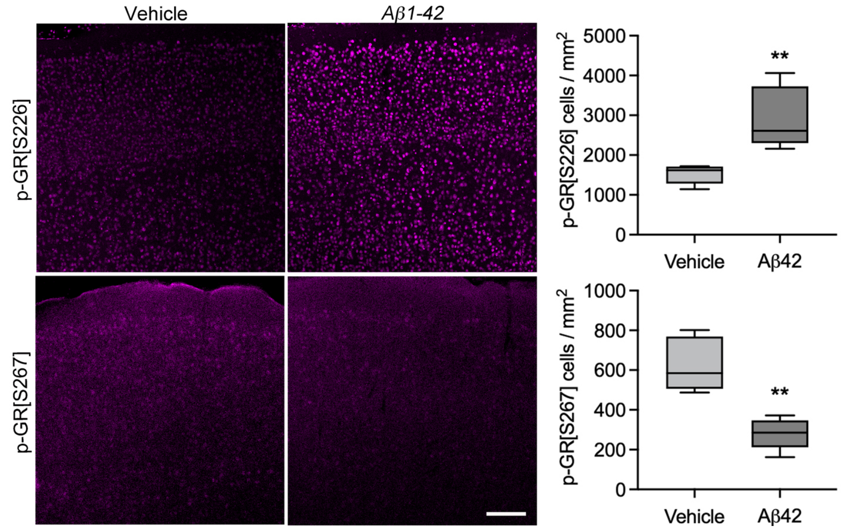

Fig. S11. GR phosphorylation status in motor cortex of mice injected with oligomers

Coronal sections of mouse cortex harvested one week after i.c.v. injection of Aβ42 oligomers and stained with antibodies against the cortisol-dependent p-GR[S226] or the BDNF-dependent p-GR[S267]. Scale =100 μm. Quartiles and median of dataset from Min-to-Max (N=5 mice/group). Mann-Whitney test for comparing vehicle and Aβ42 groups, ***P*<0.01.

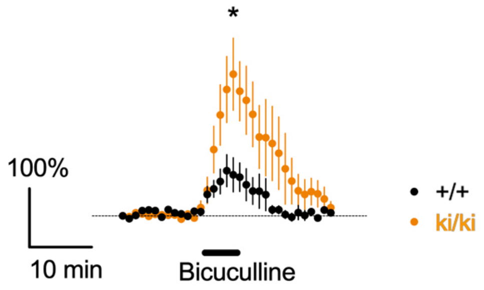

Fig. S12. Effect of GABA-A receptor antagonist on cortical field potentials in mice lacking p-GR sites.

Effect of 3.5M bicuculline on field potentials in motor cortex reversed on washout with ACSF. N=33 slices for each genotype. Mann-Whitney test comparing genotypes *p<0.05.
